# Integrated analysis of mRNA and miRNA expression profiling in rice backcrossed progenies (BC_2_F_12_) with different plant height

**DOI:** 10.1371/journal.pone.0184106

**Published:** 2017-08-31

**Authors:** Aqin Cao, Jie Jin, Shaoqing Li, Jianbo Wang

**Affiliations:** State Key Laboratory of Hybrid Rice, College of Life Sciences, Wuhan University, Wuhan, China; Dokuz Eylul Universitesi, TURKEY

## Abstract

Inter-specific hybridization and backcrossing commonly occur in plants. The use of progeny generated from inter-specific hybridization and backcrossing has been developed as a novel model system to explore gene expression divergence. The present study investigated the analysis of gene expression and miRNA regulation in backcrossed introgression lines constructed from cultivated and wild rice. High-throughput sequencing was used to compare gene and miRNA expression profiles in three progeny lines (*L1710*, *L1817* and *L1730*), with different plant heights resulting from the backcrossing of introgression lines (BC_2_F_12_) and their parents (*O*. *sativa* and *O*. *longistaminata*). A total of 25,387 to 26,139 mRNAs and 379 to 419 miRNAs were obtained in these rice lines. More differentially expressed genes and miRNAs were detected in progeny/*O*. *longistaminata* comparison groups than in progeny/*O*. *sativa* comparison groups. Approximately 80% of the genes and miRNAs showed expression level dominance to *O*. *sativa*, indicating that three progeny lines were closer to the recurrent parent, which might be influenced by their parental genome dosage. Approximately 16% to 64% of the differentially expressed miRNAs possessing coherent target genes were predicted, and many of these miRNAs regulated multiple target genes. Most genes were up-regulated in progeny lines compared with their parents, but down-regulated in the higher plant height line in the comparison groups among the three progeny lines. Moreover, certain genes related to cell walls and plant hormones might play crucial roles in the plant height variations of the three progeny lines. Taken together, these results provided valuable information on the molecular mechanisms of hybrid backcrossing and plant height variations based on the gene and miRNA expression levels in the three progeny lines.

## Introduction

Rice (*Oryza sativa*), one of the most important food crops, feeds a large number of the world’s population. With the availability of reference genome sequences, rice has also become an eminent model system in plant research for monocotyledon species. The genus *Oryza*, including 2 cultivated species (*O*. *sativa* and *O*. *glaberrima*) and 22 wild species, was classified into 10 genome types, including the AA, BB, CC, BBCC, CCDD, DD(EE), FF, GG, HHJJ and HHKK genomes [1]. The AA genome contains 8 species in the genus *Oryza*, consisting of 2 cultivated species and 6 wild species (*O*. *meridionalis*, *O*. *rufipogon*, *O*. *nivara*, *O*. *glumaepatula*, *O*. *barthii* and *O*. *longistaminata*). The wild species in the genus *Oryza* are a valuable source of novel genetic variation, and the majority of genetic variation in the genus *Oryza* remains untapped [2]. *O*. *sativa* includes 2 primary groups, the *indica* and *japonica* subspecies. Rice cultivar 9311, an elite parental line of *indica*, was used to develop super hybrid rice in China, such as cultivar *LYP9* [3]. *O*. *longistaminata*, a native to African rhizomatous perennial wild rice species, potentially diverged from *O*. *glaberrima* approximately 1.9 MYA [4]. Additionally, *O*. *longistaminata* contains many potentially useful characters, such as strong rhizomes, and strong resistance to stress [5, 6]. Several genes, such as *Rhz2*, *Rhz3* and *Xa21*, have been identified and used for developing perennial rice and blight disease resistance rice [5, 7].

Hybrid rice with a combination of divergent genomes, leading to instant and profound genome modifications, has been effectively utilized to increase rice production based on their novel phenotypes, such as grain yield, stress tolerance, biomass production and plant height. As the majority of genetic variation primarily existed in wild species of the genus *Oryza* [2], the introduction of novel genes or gene-networks into cultivated rice from the wild species is an effective strategy to increase rice yield. To date, many backcross introgression lines (BILs), introgression lines (ILs) and near-isogenic lines (NILs) have been developed to introduce novel characters from the wild or resistant species into the elite breeding material in many crop species [8, 9]. Two sets of BC_7_F_2_ lines derived from RD23 (*O*. *sativa*) and *O*. *longistaminata* were generated to examine hybrid sterility and plant height, using genetic maps and molecular markers [9]. To detect the grain mineral concentration of of BILs in two environments, 2 elite *indica* varieties were crossed with a Zn-dense rice variety and 2 sets of BILs were raised [8]. A BC_4_F_2_ population of ILs was constructed to identify and map a novel blast resistance gene using SSR markers [6]. Studies on metabolic and gene expression levels in BILs and NILs have recently emerged. For example, to explore the impact of the introgression segment on a rice growth, the metabolic profiling and physiological trait analyses were performed between IL and its recurrent parent [10]. However, the roots and leaves of 2 pairs of NILs (drought-tolerant and susceptible rice) were used to unravel drought tolerant genes under different drought stress conditions through transcriptome analysis [11, 12]. Moreover, several genotype-specific drought-induced genes and higher expressed genes were identified as contributors to drought tolerate using comparative transcriptome sequencing [13]. Therefore, the BILs and ILs of rice are invaluable resources for rice genetic improvement, particularly those with wild rice as the donor parent. These studies on BILs and ILs of rice primarily focused on stress resistance but rarely examined plant height.

Micro-RNAs (miRNAs) of 20–30 nt are endogenously expressed short non-coding small RNAs (sRNAs) affecting gene expression through post-transcriptional mechanisms, similar to other classes of sRNAs. When miRNAs near-perfectly complement to their target mRNAs, target gene expression will be disturbed, or mRNA translation will be inhibited in plants [14]. To date, many miRNA studies have been conducted, and the essential roles of miRNAs in many biological processes, including various metabolic pathways, signal transduction and stress responses, have been characterized in plants [15–19]. In plant species, most miRNAs are highly conserved, but the expression levels of non-conserved miRNAs are lower, which is difficult to examine using small-scale sequencing projects. Recently, high throughput sequencing as an effective method have identified and estimated the expression profiles of miRNAs in different plant tissues [3, 18, 20, 21], and many low abundant and novel non-conserved miRNAs have been identified. In the rice miRBase, 713 miRNAs have been identified, including those associated with plant architecture [22], stem and grain development [20, 23].

Recently, studies on the integrated analysis of mRNA and miRNA expression profiling have been reported [18, 19, 21, 24–27]. A comprehensive interaction analysis of miRNA and mRNA revealed the regulatory mechanisms of plant height in *Gossypium hirsutum* [19], provided small RNA-mediated regulation mechanisms in hexaploid wheat [21], and identified a number of important regulators in tomato during *Alternaria*-stress responses [27]. The analysis of miRNAs and mRNAs expression levels between *indica* and *japonica* rice subspecies revealed that the target gene expression was more easily repressed by highly conserved miRNAs than by lowly conserved miRNAs [18]. However, little effort has been made toward exploring the mRNA and miRNA expression diversity of ILs rice and their parents to study the molecular mechanism associated with phenotypic changes, particularly plant height.

In the present study, next-generation high-throughput sequencing technology and bioinformatics were utilized to present an integrative analysis of the mRNA and its regulatory miRNA networks in three progeny lines of BILs and their parents. We focused on the following: (1) we examined mRNAs and miRNAs expression level changes in three progeny lines; (2) we determined whether the differentially expressed mRNAs and miRNAs in the three progeny lines were influenced by genome composition; and (3) we conducted an integrative analysis of the mRNA and miRNA regulation network in three progeny lines and their parents, which was essential to clarify the modulation of gene expression at the post-transcriptional level. These results will provide a comprehensive assessment of mRNA and miRNA expression levels, thereby increasing the current knowledge of the complex molecular mechanism of gene introgression that resulting in plant height variations in the three progeny lines.

## Materials and methods

### Plant materials and growth conditions

Three progeny lines of backcrossed introgression lines (BC_2_F_12_) and their parents, *O*. *longistaminata* and *O*. *sativa* ssp. *indica* cv. 9311, were used as plant materials in the present study (all plant materials were provided by State Key Laboratory of Hybrid Rice, Wuhan University, Wuhan, China). BC_2_F_12_ (containing 152 lines) were constructed from *O*. *sativa* as the female crossed with *O*. *longistaminata* to generate F1 hybrid; later, F1 as male was backcrossed with *O*. *sativa* to develop BC_1_F_1_, and 65 BC_1_F_1_ individuals were for backcrossing again with *O*. *sativa* to develop BC_2_F_1_ plants and subsequently BC_2_F_1_ using a single seed descent method by 11 generation self-fertilization to produce BC_2_F_12_. Three progeny lines, namely *L1710*, *L1817* and *L1730*, with different plant height were used in subsequent studies. Most of the chromosome complements of the three progeny lines were inherited from *O*. *sativa*, and a fraction of the complements were inherited from *O*. *longistaminata* (15.72% in *L1710*, 10.32% in *L1817* and 16.42% in *L1730*), which were calculated based on their SNP loci (unpublished, S1 Fig). Additionally, heterozygosity chromosome complements were most abundant in *L1817* among the three progeny lines. Germinated seeds of the three progeny lines and *O*. *sativa* were sown in soil, and at approximately 30 days old, the seedlings were transplanted into plots in the greenhouse. The wild species *O*. *longistaminata* is a perennial plant, and rhizomes are essential organs for its rapid growth at the beginning of the next growing season. The rhizomes of *O*. *longistaminata* were planted in plots and clonally propagated in the same greenhouse.

### Histological observation

For histological examination, the third internodes of natural growing plants, *L1710*, *L1817*, *L1730*, *O*. *sativa* and *O*. *longistaminata*, were collected and fixed with FAA (formalin: glacial acetic acid: 50% ethanol = 1: 1.2: 17.8) solution. After being dehydrated in a graded ethanol series and being rendered transparent in a grade transparent agent series, internodes were embedded in paraffin. Subsequently, 10 μm thick sections were sectioned on a rotary microtome (Leica Biosystems, Nussloch, Germany) and placed onto glass slides. The tissue sections were observed under a light microscope (BX51; Olympus, Tokyo, Japan), and the cell lengths were measured, after Safranine (Sinopharm Chemical Reagent Co., Ltd, Shanghai, China) and Fast Green staining (Sigma Aldrich, USA).

### Construction of mRNA and small RNA libraries

Stem tissue from five individual plants of each line at jointing-booting stage was collected and immediately stored in liquid nitrogen for total RNA extraction. Total RNAs from each line were separately extracted from the five individual stem tissues using TRIzol reagent (Invitrogen, Carlsbad, CA, USA) and purified using RNase-free DNase I (Fermentas, MD, USA), and the mRNAs were used to synthesize cDNAs, which were subsequently qualified and quantified, as previously described [28]. Equal amounts of cDNA from five individual plants of the same line were combined into one library for sequencing using the Illumina HiSeq 2000 platform.

Total RNA extraction for constructing a small RNA sequencing library was similar to that of mRNA library. The sRNA selection, library construction and sequencing were performed as previously described [15].

### Analysis of mRNA and small RNA sequencing data

Illumina sequencing, data processing and RNA sequencing generated read mapping to the reference genome (the Rice Genome Annotation Project, http://rice.plantbiology.msu.edu) were performed as previously described [29]. The Illumina HiSeq2000 sequencing, data processing and small RNA sequencing generated read mapping to the same reference genome were followed as previously described [30].

### Evaluations of genes in RNA-Seq data and miRNAs in small RNA-Seq data

Using ERANGE software (version 4.0) (http://woldlab.caltech.edu/gitweb/), we evaluated the expression of genes in RNA-Seq data by assigning reads to rice reference genomes. The gene expression levels were normalized in terms of RPKM [31].

The miRNA read count was normalized to TPM (transcripts per million) using the formula: normalized expression = actual miRNA count/total count of clean reads×1,000,000.

To group all annotated genes/miRNAs from the three progeny lines and their parents with similar expression patterns, hierarchical cluster analysis was performed. The cluster analysis of genes and miRNAs were conducted using the Heatmap and Cluster 3.0 software with Pearson correlation as the distance measure, and the Tree view tool was used to visualize results [32].

### Analysis of differentially expressed genes and miRNAs

Differential expression analysis was performed using R packages of DESeq [33]. The false discover rate (FDR) was used to correct and determine the threshold of the P value for multiple comparisons. In the present study, genes that exhibited fold changes ≥5 (log_2_ Ratio ≥2.322) and FDR ≤0.001 were defined as differentially expressed genes (DEGs). The miRNAs with an absolute value of log_2_ Ratio ≥1 and P value ≤0.05 were determined to show significant differential expression.

### Functional annotation analysis of genes and miRNA target genes

All miRNA sequences from five lines were mapped to the rice reference genome (http://rice.plantbiology.msu.edu). The criteria for target predicting used in the present study were previously described [34, 35].

Gene ontology (GO) annotation (http://www.blast2go.org/) of all expressed genes and all predicted target genes of miRNAs were performed using the Blast2GO program (version 2.3.5). A web gene ontology annotation plot (WEGO) server (http://wego.genomics.org.cn/cgi-bin/wego/index.pl) was utilized for GO functional classification.

In the KEGG (Kyoto Encyclopedia of Genes and Genomes) pathway analysis, all DEGs were mapped to the KEGG database terms. Based on the RPKM of the genes, we identified up/down-regulated pathways in the three progeny lines compared with their parents. The results were analyzed and visualized using Cytoscape software (version 2.6.2) (www.cytoscape.org/).

### Real-time quantitative PCR validation (qRT-PCR)

To validate the accuracy of the Illumina sequencing, 12 genes and 6 miRNAs were randomly selected for qRT-PCR reactions. In addition, the expressions of 6 differentially expressed miRNAs and their predicted target genes were validated by qRT-PCR reactions. Total RNAs were extracted from the plant stems, and oligo (dT) was used to reverse transcribe the RNAs of genes, and specific primers for RT-qPCR were designed using the Primer 5.0 software (Premier Biosoft International, Palo Alto, CA, USA, http://www.premierbiosoft.com/index.html). Since miRNAs are short and without polyA tails, the reverse-transcription of miRNAs was completed using a miRNA-specific stem-loop primer, according to stem-loop primer designing rules [36]. To normalize each gene threshold cycle reaction, *actin1* was used as internal reference gene, and the U6 snRNA was selected as an internal control for miRNA expression. The qRT-PCR reaction was performed as previously described [15, 29]. The validation analysis of genes and miRNAs were performed in triplicate with three biological replicates. All primer sequences used in the present study were listed in the S1 and S2 Tables.

## Results

### Comparison of stem traits among the three progeny lines and their parents

**Fig 1:**
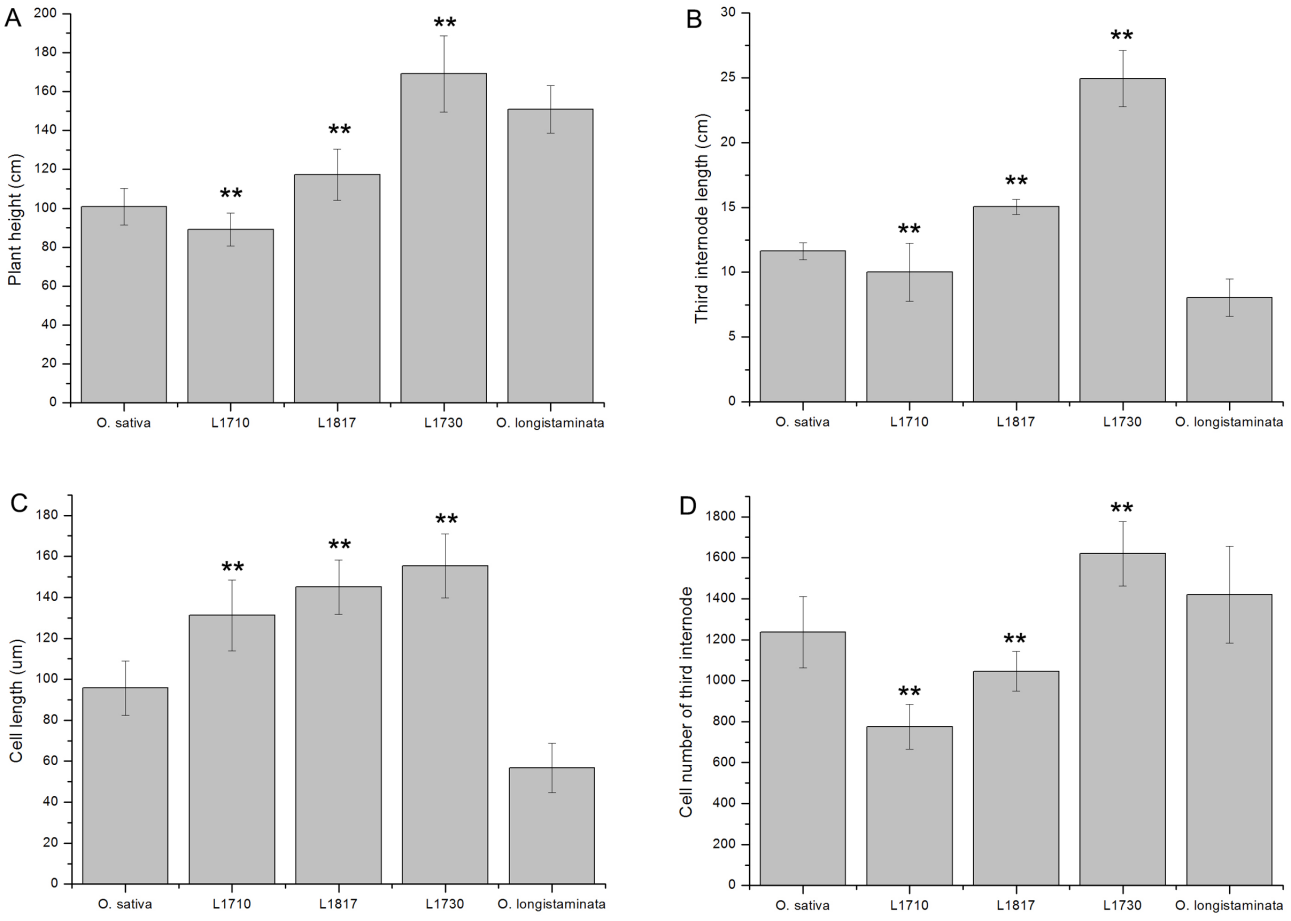
Morphological phenotypes characterizations of the three progeny lines and their parents. (A) The rice height of five lines. (B) The third internode length of five lines. (C) The cell length of third internode in five lines. (D) The cell number of third internode in five lines. Asterisks indicate a significant difference (P< 0.01) between progeny line and their parents through t-test.

In the present study, the three progeny lines (*L1710*, *L1817* and *L1730*) of a backcrossed introgression line (BC_2_F_12_), recurrent parent *O*. *sativa* and donor parent *O*. *longistaminata* were utilized. Compared with their parents, the plant height of *L1730* was taller, and *L1710* was shorter, while *L1817* was taller than *O*. *sativa*, but shorter than *O*. *longistaminata* (Fig 1a). Rice plant height was primarily dependent on stem length, which was determined based on the number and length of internodes. In the present study, we observed that the total number of elongated internodes was the same in *L1710*, *L1817*, *L1730* and *O*. *sativa*. Additionally, changes in the first, second, third, fourth, and fifth (uppermost) internode lengths of the three progeny lines and *O*. *sativa* showed similar trends. However, the number and length of internodes were unsteady in *O*. *longistaminata*. For the third internode length, *L1817* and *L1730* were longer than their parents, while *L1710* was longer than *O*. *longistaminata* but shorter than *O*. *sativa* (Fig 1b). Internode elongation resulted from the cell division in the meristem and the cell elongation in the elongated region. To investigate whether variations of internode length were primarily attributed to the cell elongation and/or the cell division, the longitudinal sections of the third internodes of five lines were subjected to microscopic observation. Surprisingly, the cell length of three progeny lines was longer than that of their parents (Fig 1c). Among three progeny lines, the cell length of *L1730* was longest, *L1710* was shortest and *L1817* was intermediate. The results showed that the cell length of the three progeny lines was consistent with their plant height variations. After calculating the cell number (internode length divided by the cell length of corresponding internode), we observed decreased cell number in *L1710* compared with their parents, decreased cell number in *L1817* compared with *O*. *longistaminata*, and increased cell number in *L1730* compared with *O*. *sativa* (Fig 1d). These results showed that the increased cell length and cell number were important determinants of internode length in *L1730*, while *L1817* internode length was primarily caused by increased cell length. However, the reduced cell number and increased cell length determined the internode length of *L1710*. To investigate the complex regulation of gene expression and characterize the various biological processes contributing to changes in plant architecture in the progeny lines, the gene and miRNA expression levels were characterized.

### Analysis of gene expression in the progeny lines and their parents

To elucidate the gene expression patterns of stem in the three progeny lines and their parents, internodes at jointing-booting stage were used to construct RNA-Seq libraries sequenced using the Illumina HiSeq 2000 platform. After filtering low-quality reads and adaptor sequences, more than 11 million clean reads (11,959,710 in *L1710*, 12,636,297 in *L1817*, 11,832,864 in *L1730*, 11,792,127 in *O*. *sativa* and 11,800,115 in *O*. *longistaminata*) were obtained in each library constructed from these rice lines (S3 Table). Using SOAP aligner/soap2 software, 77.75% to 87.89% of all reads were matched to the reference genome, with no more than two base mismatches allowed in the alignment.

**Fig 2:**
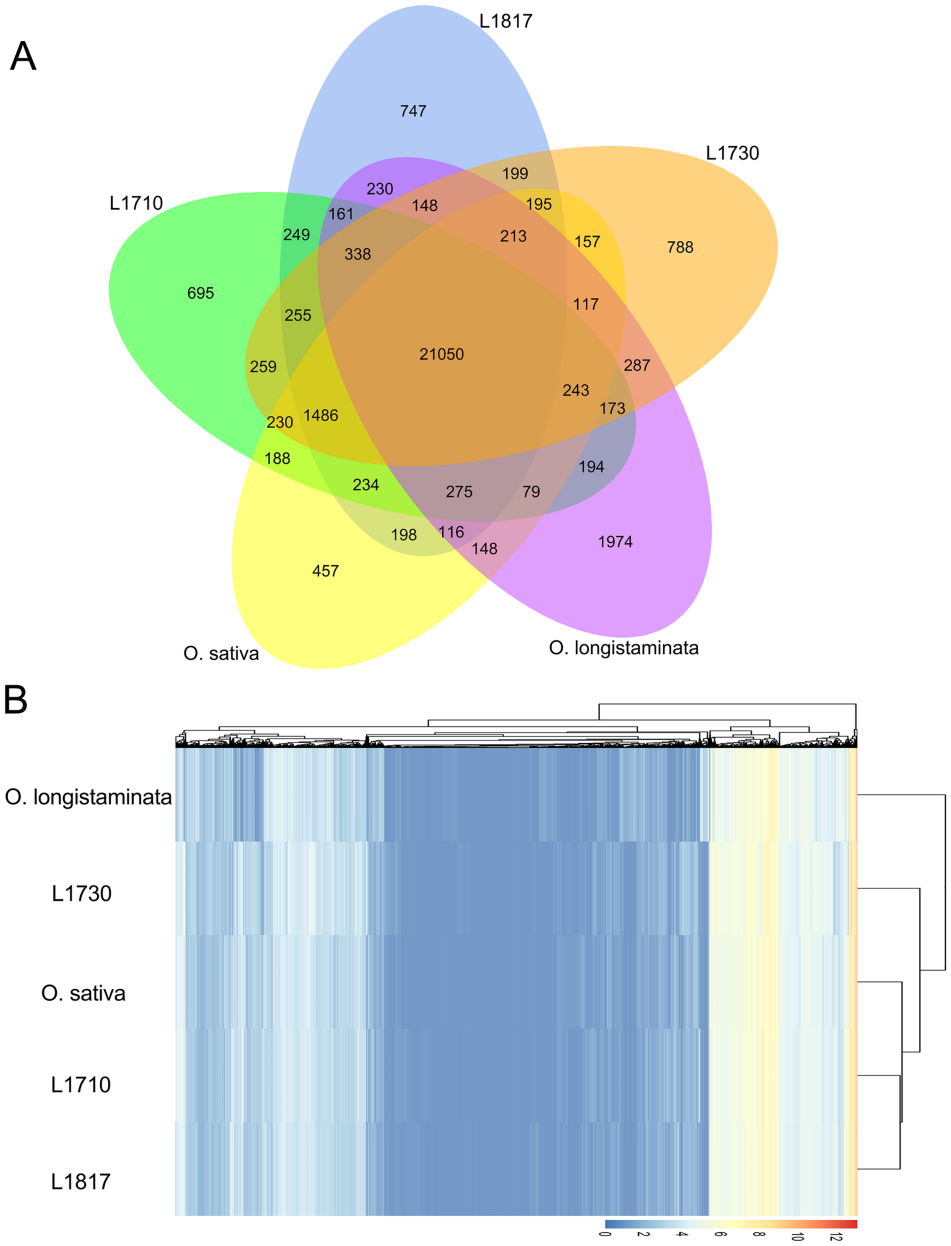
Venn diagram analysis and hierarchical cluster analysis in the five lines. (A) Venn diagram analysis of co-expressed and specially expressed genes in the five lines. (B) Hierarchical cluster analysis of all gene models in five lines. The branch length indicates the degree of variance, and the color represents the logarithmic intensity of expressed genes. Lines groups are shown as columns, individual expressed genes are arrayed in rows.

In the present study, a total of 26,110, 26,094, 26,138, 25,386 and 25,746 expressed genes were identified in the stems of *L1710*, *L1817*, *L1730*, *O*. *sativa* and *O*. *longistaminata*, respectively. Among the identified genes, 24,140 genes were longer assembled sequences, with gene lengths exceeding 1,000 bp (S4 Table). The Venn diagram showed the distribution of the expressed genes in the five lines. We observed that 21,050 (65.61%) genes were commonly expressed (co-expressed) in the five lines, and the number of co-expressed genes was obviously much higher than the number of specifically expressed genes (Fig 2a). In addition, the specifically expressed genes were most abundant in *O*. *longistaminata* (6.15%) and least abundant in *O*. *sativa* (1.42%) among the five lines. The results suggested that these co-expressed genes might act as housekeeping genes during rice growth and development stage.

Heat maps for cluster analysis were used to investigate correlations among the 5 transcriptome profiles. In the present study, *L1710* and *L1817* were closely grouped together, and subsequently they were close to *O*. *sativa*, followed by *L1730* (Fig 2b). Additionally, the gene expression profile of *L1730* was closer to *O*. *longistaminata* than the other lines. The results of all gene expression profiles reflected that most of the gene expression patterns were closer to the recurrent parent in the three progeny lines, which might be influenced by their similar chromosome complements.

### Differentially expressed genes (DEGs) among five lines

Many molecular mechanisms were involved in the reprogramming of interacting genomes in backcrossed progeny, and DEGs were key elements influencing the plant morphology changes. Interestingly, the three progeny lines, the nuclear genetic backgrounds of which were primarily inherited from *O*. *sativa*, showed obvious phenotypic variations in plant height. Thus, the present study was focused on stem gene expression patterns among five lines to explore genes, which may play crucial roles in progeny phenotypic variations.

**Fig 3:**
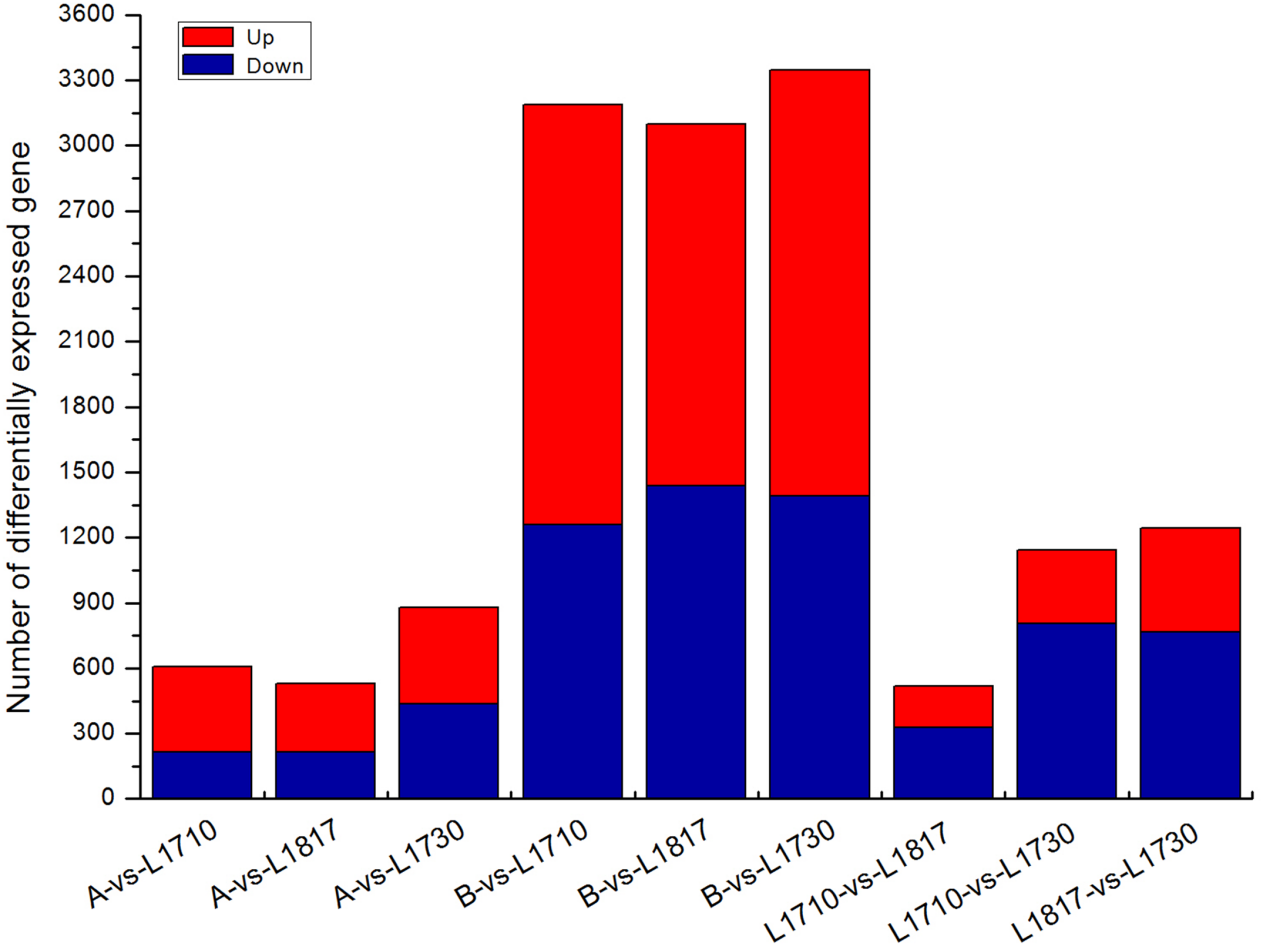
Differentially expressed genes (DEGs) in three progeny lines. A and B stand for *O*. *sativa* and *O*. *longistaminata*, respectively.

DEGs were analyzed to determine the phenotypic diversity at the molecular level among backcrossed progeny lines. Putative DEGs were identified using the following criteria: (1) the absolute value of the log_2_ ratio ≥2.322; and (2) P value ≤0.001 and FDR (false discovery rate) ≤0.001. In the present study, the DEGs in three progeny line/*O*. *longistaminata* comparison groups exceeded that in three progeny line/*O*. *sativa* comparison groups (Fig 3). For example, 3,188 DEGs were identified in the *L1710*/*O*. *longistaminata* comparison group, while only 605 DEGs were identified in the *L1710*/*O*. *sativa* comparison group. Among all comparison groups, the number of DEGs was the least in *L1817* compared with their parents, which might reflect an additional heterozygous genome in *L1817* than in the other lines. Additionally, more than 50% of the DEGs were up-regulated in the three progeny lines compared with their parents. Notably, most of the DEGs were down-regulated in the higher plant line in the comparison groups among the three progeny lines (Fig 3).

### GO analysis of DEGs in three backcrossed progeny lines

**Fig 4:**
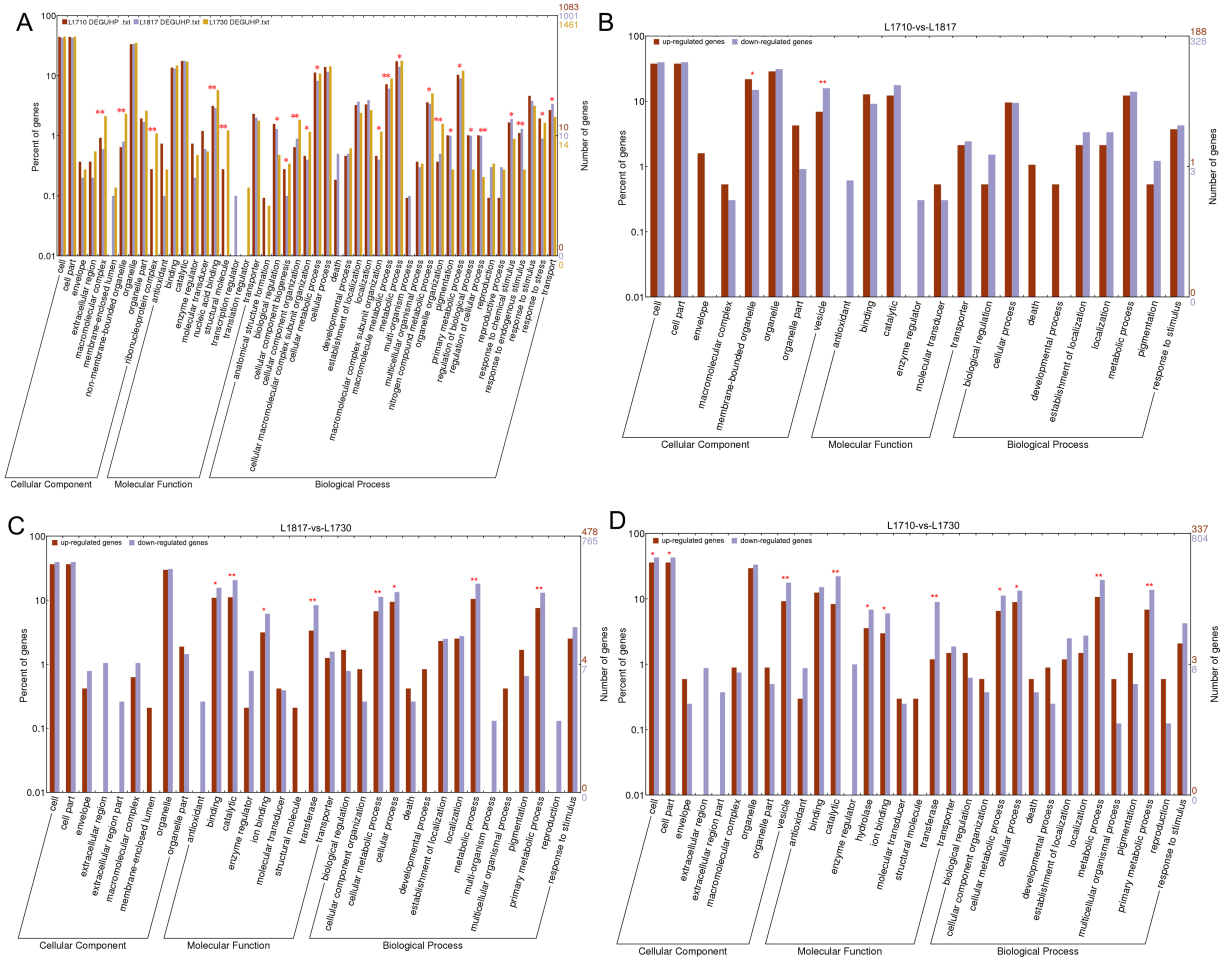
GO analysis of DEGs in the three progeny lines. (A) GO analysis of DEG_UHP_ in the three progeny lines. GO analysis of up/down-regulated DEGs in the *L1710*/*L1817* comparison group (B), the *L1710*/*L1730* comparison group (C) and the *L1817*/*L1730* comparison group (D). The x-axis represents the name of the GO subcategories. The right y-axis indicates the number of genes expressed in a given sub-category. The left y-axis indicates log (10) scale, the percent of a specific category of genes in that main category. GO terms with P value<0.05 were denoted by one star, GO terms with P value<0.01 were denoted by two stars.

For a detailed analysis, DEGs between O. *sativa* and *O*. *longistaminata* were designated DEG_PP_, and the DEGs between progeny and their parents were designated DEG_HP_. For the DEG_HP_ contained 2 classes, DEG_O_ (shared by DEG_HP_ and DEG_PP_) and DEG_UHP_ (uniquely belonging to DEG_HP_), and DEG_UHP_ might play roles in phenotype changes between progeny lines and their parents, hence we primarily focused on DEG_UHP_. In total, more DEG_UHP_ was discovered in *L1730* (1,461) than that in *L1710* (1,083) and *L1817* (1,001). GO analysis of DEG_UHP_ on the WEGO website (http://wego.genomics.org.cn/cgi-bin/wego/index.pl) among the three progeny lines was performed to reveal important GO terms of three categories (cellular component, molecular function and biological process). We identified 23 GO terms in the three progeny lines that exhibited statistically significant differences (P <0.05), such as term ‘metabolic process’, ‘cellular process’, ‘organelle organization’, ‘regulation of cellular process’, ‘transport’, ‘biological regulation’ and ‘primary metabolic process’ (Fig 4a). Genes participating in these terms showed differential expression patterns. For example, *OsXTH8* (LOC_Os08g13920) involved in ‘primary metabolic process’ was down-regulated in *L1817* and *L1730* compared with *O*. *sativa*, but up-regulated in *L1710* and *L1817* compared with *O*. *longistaminata*. Moreover, 2 terms in cellular component category, ‘ribonucleoprotein complex’ and ‘non-membrane-bounded organelle’, 2 terms in molecular function category, ‘structural molecule activity’ and ‘nucleic acid binding’, and 6 terms in biological process category, including terms ‘cellular component organization or biogenesis’, ‘macromolecular metabolic process’, ‘organelle organization’, ‘regulation of cellular process’, ‘biological regulation’ and ‘response to endogenous stimulus’, with the P <0.01, might contribute to plant height variation in the three progeny lines.

GO analysis of up/down-regulated DEGs between each progeny line was performed to identify the functional category that the DEGs were primarily involved in. As a result, 2 terms in the *L1710*/*L1817* comparison, 8 terms in the *L1817*/*L1730* comparison and 11 terms in the *L1710*/*L1730* comparison showed statistically significant differences (Fig 4b–4d). Among these terms, 3 terms of molecular function category (‘catalytic activity’, ‘ion binding’ and ‘transferase activity’) and 4 terms of biological process category (‘cellular process’, ‘cellular metabolic process’, ‘metabolic process’ and ‘primary metabolic process’) showed statistically significant differences in the *L1710*/*L1730* comparison and the *L1817*/*L1730* comparison. In addition, ‘vesicle’ showed statistically significant differences in the *L1710*/*L1730* comparison and the *L1710*/*L1817* comparison. Furthermore, ‘cell’, ‘cell part’ and ‘hydrolase activity’ in the *L1710*/*L1730* comparison, ‘membrane-bounded organelle’ in the *L1710*/*L1817* comparison and ‘binding’ in the *L1817*/*L1730* comparison showed statistically significant differences. In ‘metabolic process’, β-1,3-glucanase gene (*Osg1*, LOC_Os01g71930) was up-regulated in *L1730* compared with *L1817* and *L1710*. *OsUGE1* (LOC_Os05g51670) involved in ‘vesicle’ and ‘primary metabolic process’ was down-regulated, while *OsPIN10b* (LOC_Os05g50140) involving in ‘vesicle’ was up-regulated in *L1730* compared with *L1817* and *L1710*. *OsEXP1* (LOC_Os04g15840) and *CYP714B1* (LOC_Os07g48330) involved in ‘vesicle’ were up-regulated in *L1817*. According to the above results, we speculated that the genes related to ‘vesicle’, ‘catalytic activity’, ‘ion binding’, ‘transferase activity’, ‘cellular process’, ‘cellular metabolic process’, ‘metabolic process’ and ‘primary metabolic process’ might contribute to the changes in plant height in the three progeny lines at the jointing stage.

### KEGG pathway analysis of DEGs in the three progeny lines

Since genes typically interacted with other genes participating in certain biological functions, the DEGs were mapped to reference canonical pathways in the KEGG (http://www.genome.ad.jp/kegg/). KEGG pathway analysis was used to provide information to further understand the biological function of genes during rice growth process and identify the genes involved in significantly enriched pathways. All DEGs were assigned to more than 120 pathways in three progeny line/*O*. *longistaminata* comparison groups, while approximately 90 pathways were detected in three progeny line/*O*. *sativa* comparison groups (S5 Table). Most pathways were up-regulated in *L1710* and *L1817* compared with their parents, while more down-regulated pathways were observed in *L1730*. Furthermore, approximately 15% to 19% pathways were significantly enriched pathways (P ≤0.05) in the three progeny lines compared with *O*. *sativa*, which were fewer than that in three progeny line/*O*. *longistaminata* comparison groups (21–32%). Compared with progeny line/*O*. *sativa* comparison groups, more KEGG pathways and significantly enriched pathways existed in progeny line/*O*. *longistaminata*, which might be caused by more DEGs existed in three progeny line/*O*. *longistaminata* comparison groups.

**Fig 5:**
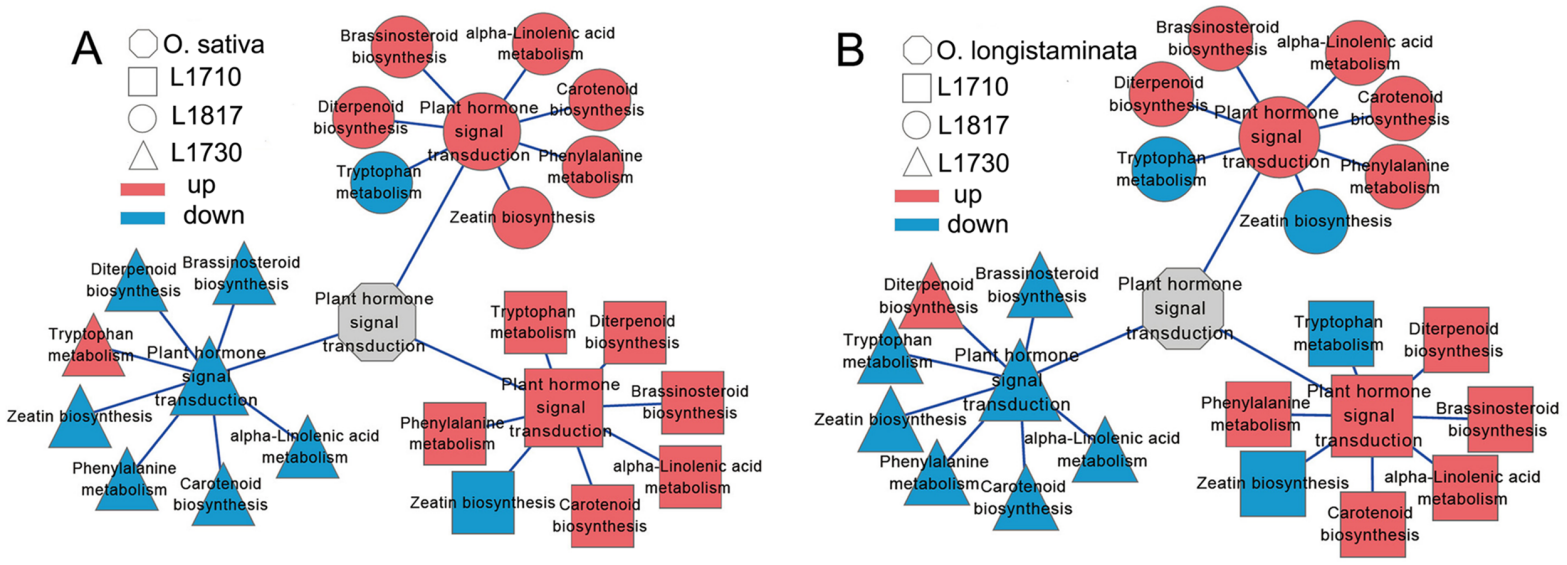
Pathway of plant hormone signal transduction analysis in the three progeny lines. The up/down regulation of plant hormone signal transduction pathway in the progeny/*O*. *sativa* comparison groups (A) and the progeny/*O*. *longistaminata* comparison groups (B). Red standing for up-regulated and blue standing for down-regulated.

In addition, we pursued top 10 up/down-regulated pathways based on the RPKM of DEGs in three progeny lines. The results showed that ‘phenylalanine metabolism’ was up-regulated in *L1710* and *L1817*, and down-regulated in *L1730* (Table 1). The pathway ‘ribosome’ was up-regulated in most comparison groups, except in the *L1710*/*O*. *longistaminata* comparison group. Another pathway, ‘phenylpropanoid biosynthesis’ was down-regulated in *L1730* compared with *O*. *sativa*, but was up-regulated in *L1710* and *L1817*. In total, significantly enriched pathways (P ≤0.05) accounted for approximately 50% of the 20 pathways (top 10 up-regulated pathways and top 10 down-regulated pathways) in the *L1710*/*O*. *sativa* comparison group and *L1730*/*O*. *longistaminata* comparison group. In KEGG pathways, ‘plant hormone signal transduction pathway’ contained 7 secondary pathways, including ‘alpha-linolenic acid metabolism’, ‘brassinosteroid biosynthesis’, ‘carotenoid biosynthesis’, ‘diterpenoid biosynthesis’, ‘phenylalanine metabolism’, ‘tryptophan metabolism’ and ‘zeatin biosynthesis’ (Fig 5). We observed that this pathway was up-regulated in *L1710* and *L1817* compared with their parents, while its secondary pathways, ‘zeatin biosynthesis’ in *L1710* and ‘tryptophan metabolism’ in *L1817*, were down-regulated. Additionally, ‘tryptophan metabolism’ in *L1710* and ‘zeatin biosynthesis’ in *L1817* were down-regulated compared with *O*. *longistaminata*. In contrast to *L1710* and *L1817*, this pathway was down-regulated in *L1730* compared with their parents, while ‘tryptophan metabolism’ was up-regulated in the *L1730*/*O*. *sativa* comparison group. In addition, we observed that ‘diterpenoid biosynthesis’ was up-regulated in the three progeny lines compared with *O*. *longistaminata*. The genes associated with plant hormone might play critical roles in the growth and development of the progeny lines. The up-regulation and down-regulation of these pathways were closely associated with the expression level changes of many genes. In the present study, most gene expression patterns were determined to be consistent with their pathway variations. For example, *GH3* (LOC_Os07g40290) was up-regulated in *L1710* and *L1817* compared with their parents, but down-regulated in *L1730* compared with *L9311*. Additionally, *OsGSR1* (LOC_Os06g15620) and *D62* (LOC_Os06g03710) were up-regulated in the three progeny lines.

**Table 1:** Top 10 up/down-regulated KEGG pathways in the three progeny lines.

	Up-regulated pathways		Down-regulated pathways		Up-regulated pathways		Down-regulated pathways		Up-regulated pathways		Down-regulated pathways	
	A-vs-L1710	B-vs-L1710	A-vs-L1710	B-vs-L1710	A-vs-L1817	B-vs-L1817	A-vs-L1817	B-vs-L1817	A-vs-L1730	B-vs-L1730	A-vs-L1730	B-vs-L1730
**1**	**Biosynthesis of secondary metabolites (509.0)**	Metabolic pathways (1,394.3)	**Photosynthesis—antenna proteins (-2,221.5)**	Photosynthesis—antenna proteins (-809.3)	**Biosynthesis of secondary metabolites (1,052.9)**	**Plant hormone signal transduction (591.3)**	Glyoxylate and dicarboxylate metabolism (-769.3)	Histidine metabolism (-1,290.5)	Ribosome (2,216.3)	**Ribosome (2,824.4)**	Metabolic pathways (-2,502.9)	**Biosynthesis of secondary metabolites (-3,920.3)**
**2**	**Phenylpropanoid biosynthesis (464.6)**	**Biosynthesis of secondary metabolites (1,126.3)**	Metabolic pathways (-1,233.0)	Protein processing in endoplasmic reticulum (-706.9)	Plant hormone signal transduction (517.1)	Carotenoid biosynthesis (521.2)	Photosynthesis (-459.4)	Biosynthesis of secondary metabolites (-925.9)	Base excision repair (214.9)	**Base excision repair (271.9)**	Biosynthesis of secondary metabolites (-1,455.7)	Metabolic pathways (-3,510.3)
**3**	**Phenylalanine metabolism (416.6)**	Glyoxylate and dicarboxylate metabolism (706.9)	**Porphyrin and chlorophyll metabolism (-729.7)**	Spliceosome (-497.2)	**Plant-pathogen interaction (475.2)**	**Plant-pathogen interaction (398.0)**	Carbon fixation in photosynthetic organisms (-136.2)	Protein processing in endoplasmic reticulum (-684.1)	**Purine metabolism (174.8)**	Oxidative phosphorylation (194.9)	**Glyoxylate and dicarboxylate metabolism (-842.8)**	Plant hormone signal transduction (-1,657.4)
**4**	**Plant-pathogen interaction (356.8)**	**Phenylalanine metabolism (637.1)**	Photosynthesis (-486.0)	Ubiquitin mediated proteolysis (-445.9)	**Carotenoid biosynthesis (377.5)**	**Phenylalanine metabolism (300.2)**	Propanoate metabolism (-112.4)	Metabolic pathways (-654.4)	Ribosome biogenesis in eukaryotes (163.2)	RNA transport (177.7)	**Phenylpropanoid biosynthesis (-570.7)**	Histidine metabolism (-1,373.8)
**5**	**Plant hormone signal transduction (352.6)**	Plant hormone signal transduction (503.6)	Carbon fixation in photosynthetic organisms (-330.7)	**Tryptophan metabolism (-338.9)**	ABC transporters (155.5)	Fatty acid eBation (213.0)	Pentose and glucuronate interconversions (-77.4)	**Pyrimidine metabolism (-583.4)**	Ubiquitin mediated proteolysis (131.8)	**Mismatch repair (159.4)**	Plant hormone signal transduction (-498.7)	**Phenylpropanoid biosynthesis (-1,301.8)**
**6**	Cysteine and methionine metabolism (346.2)	**Cysteine and methionine metabolism (490.5)**	**Glyoxylate and dicarboxylate metabolism (-212.9)**	Circadian rhythm–plant (-288.9)	**Phenylalanine metabolism (150.3)**	**Diterpenoid biosynthesis (201.6)**	Cyanoamino acid metabolism (-51.7)	**Purine metabolism (-529.7)**	Mismatch repair (124.4)	**DNA replication (154.1)**	Phenylalanine metabolism (-483.4)	**Phenylalanine metabolism (-788.8)**
**7**	Ribosome (140.0)	**Phenylpropanoid biosynthesis (443.8)**	**Circadian rhythm–plant (-207.9)**	Peroxisome (-274.2)	Fatty acid eBation (148.9)	ABC transporters (184.6)	Histidine metabolism (-36.4)	**Cyanoamino acid metabolism (-525.8)**	DNA replication (122.6)	Ribosome biogenesis in eukaryotes (150.9)	Photosynthesis—antenna proteins (-432.9)	Protein processing in endoplasmic reticulum (-691.1)
**8**	Phosphatidylinositol signaling system (109.0)	**Plant-pathogen interaction (425.0)**	Propanoate metabolism (-106.9)	**Cyanoamino acid metabolism (-270.1)**	Ribosome (141.8)	Cysteine and methionine metabolism (183.6)	Arginine and proline metabolism (-32.6)	Spliceosome (-453.5)	Nucleotide excision repair (113.4)	Homologous recombination (132.9)	Photosynthesis (-431.3)	**Pyrimidine metabolism (-601.0)**
**9**	**alpha-Linolenic acid metabolism (103.3)**	Phenylalanine, tyrosine and tryptophan biosynthesis (263.4)	Endocytosis (-34.6)	Endocytosis (-262.3)	Phenylpropanoid biosynthesis (141.5)	Glyoxylate and dicarboxylate metabolism (158.1)	Porphyrin and chlorophyll metabolism (-29.7)	Ubiquitin mediated proteolysis (-400.4)	RNA transport (111.7)	**Nucleotide excision repair (125.7)**	Endocytosis (-316.3)	**Flavonoid biosynthesis (-539.2)**
10	Stilbenoid, diarylheptanoid and gingerol biosynthesis (94.8)	**Diterpenoid biosynthesis** **(193.8)**	Zeatin biosynthesis (-34.0)	Glucosinolate biosynthesis (-222.8)	**Metabolic pathways (141.4)**	Ribosome (151.5)	**Anthocyanin biosynthesis (-28.8)**	**Tryptophan metabolism (-314.1)**	SNARE interactions in vesicular transport (91.1)	SNARE interactions in vesicular transport (125.0)	Protein processing in endoplasmic reticulum (-299.9)	**Circadian rhythm–plant (-509.0)**

The numbers in parentheses represent the RPKM value (up or down) of all genes identified in one pathway of progeny and their parents. Pathways marked with bold font indicate significantly enriched pathways (P ≤0.05). A and B stand for *O*. *sativa* and *O*. *longistaminata*, respectively.

### Parental expression level dominance analysis in three progeny lines

**Fig 6:**
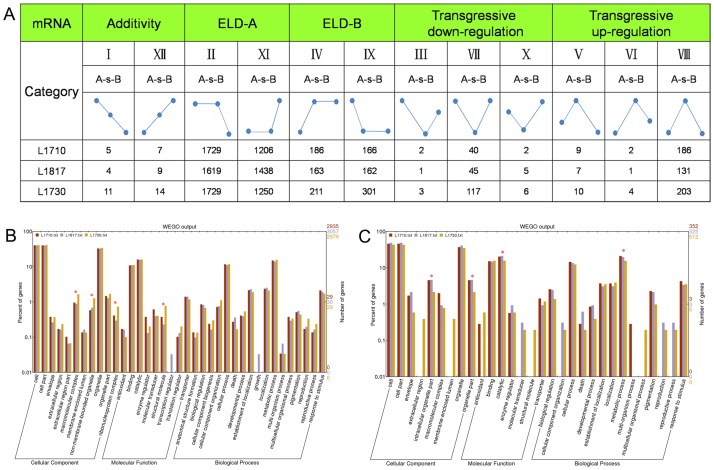
Parental expression level dominance (ELD) genes and GO analysis of ELD genes in progeny lines. (A) Twelve patterns of DEGs in the three progeny lines. GO analysis of ELD-A genes (B) and ELD-B genes (C) in the three progeny lines. GO terms with P value<0.05 were denoted by one star. ELD-A gene indicate the gene expression level in progeny is similar to that in *O*. *sativa*, but different from that in *O*. *longistaminata*; ELD-B indicate the gene expression level in progeny is similar to that in *O*. *longistaminata*, but different from that in *O*. *sativa*.

Recently, studies on hybrid progenies showed that a considerable part of genes expressed at a level approximate to that of one parent and different from that of the other parent, and have no correlation with the MPV (mid-parent value) and the additive expression. This phenomenon is defined as expression-level dominance (ELD) for the expressed genes in progenies. For a more detailed analysis, we categorized the genes into 5 major expression categories, according to previously defined criteria [37]. The 5 categories were further divided into 12 expression patterns based on the gene expression levels in the three progeny lines relative to their parents (Fig 6a). The 12 expression patterns were the expression-level dominance by *O*. *sativa* (II and XI, ELD-A), expression-level dominance by *O*. *longistaminata* (IV and IX, ELD-B), transgressive down-regulation (III, VII and X), equal to the average of *O*. *sativa* and *O*. *longistaminata* (I and XII), and transgressive up-regulation (V, VI and VIII) in the progeny line. On the whole, most genes displayed expression level dominance of one parent, and there were more ELD-A genes than ELD-B genes in the progeny lines (S6 Table). In *L1710*, we observed that the number of ELD-A genes (82.91%) was the highest, followed by ELD-B genes (9.94%) among the 5 expression categories. The proportional similarity of parental ELD genes were observed in *L1817* and *L1730*. Thus, the genes displayed ELD toward the recurrent progenitor in the three progeny lines. The proportion of ELD-B genes (9.07% to 13.27%) in the three progeny lines may be affected by their different genome composition inherited from *O*. *longistaminata* which varied from 10.32% to 16.42%. In addition, the proportion of ELD-B genes in *L1730* was higher than that in *L1817* and *L1710*, which was consistent with the plant height changes of these progeny lines.

To explore these functional differences, all parental ELD genes in the three progeny lines were assigned to the GO terms using GO functional classification analysis (WEGO). Thus, 4 GO terms of ELD genes were observed to have statistically significant differences among the three progeny lines (Fig 6b and 6c). Three terms belonging to cellular component category, i.e., ‘macromolecular complex’, ‘ribonucleoprotein complex’ and ‘non-membrane bounded organelle’, and one term of molecular function category, i.e., ‘structure molecule activity’, were statistically and significantly enriched in ELD-A genes among three progeny lines. However, ELD-B genes significantly enriched in two terms of cellular component category, ‘organelle part’ and ‘intracellular organelle part’, one term of molecular function category, ‘catalytic activity’ and one term of biological process category, ‘metabolic process’. In short, most genes in the GO analysis showed ELD-A patterns in three progeny lines, but some genes showed different patterns in different progeny lines. For example, *OsEXP1* showed ELD-A in *L1730* but showed ELD-B in *L1710* and *L1817*. ELD genes participating in these GO terms might play different roles in the growth and development at the jointing stage in the three progeny lines.

To further pursue potential functional differences between ELD-A genes and ELD-B genes in each progeny line, GO analysis was performed (S2 Fig). In *L1710*, ELD-A genes and ELD-B genes enriched in 11 GO terms that showing statistically significant differences (P<0.05). In addition, we discovered ELD-A genes and ELD-B genes enriched in 15 GO terms in *L1817* and 6 GO terms in *L1730*. Three GO terms, ‘non-membrane-bounded organelle’, ‘establishment of localization’ and ‘localization’ were only statistically significant differences in *L1730*. Moreover, we observed three GO terms (‘organelle’, ‘intracellular organelle part’ and ‘oxidoreductase activity’) in *L1710* and one GO term (‘organelle’) in *L1817* showed statistically significant differences, with P<0.01. Cellulose synthase subunit genes: *OsCESA4* (LOC_Os01g54620), *OsCESA7* (LOC_Os10g32980) and *OsCESA9* (LOC_Os09g25490) in ‘glucan biosynthetic process’ showed ELD-A pattern. Some genes showed the same expression bias in all three progeny lines, for example, *OsGI* (LOC_Os01g08700) and *OsCKX4* (LOC_Os01g71310) showed ELD-B pattern in three progeny lines. These genes showed different ELD patterns in these GO terms might differentially affect the cell growth and development.

### Identification and expression analysis of miRNAs in five lines

Five small RNA libraries from the stem materials identical to the gene expression research were constructed, and the expression of sRNAs was detected using Illumina high-throughput sequencing technology. In total, nearly 58.74 million sRNA sequencing reads were generated in five libraries. After removing adaptor contaminations and low-quality reads, 58.25 million reads were identified as small RNA (S7 Table). The length distributions of sRNAs were similar in each of the five lines. Most fragment lengths were distributed between 21 and 24 nt (S3 Fig), and these results were consistent with previous studies that 24-nt sRNAs were most abundant [15, 19–21]. Subsequently, we mapped all clean reads to small RNA databases, and these reads were clustered into 10 RNA classes (including miRNA, rRNA, snRNA, snoRNA, siRNA, piRNA, tRNA, repeat associated sRNA and degraded tags of exon or intron) and unannotated group (S7 Table). For these small RNA reads, 1,285,091 (*L1710*), 762,856 (*L1817*), 757,171 (*L1730*), 1,323,683 (*O*. *sativa*) and 1,274,873 (*O*. *longistaminata*) were identified as miRNA reads.

A total of 513 miRNAs (419, 411, 411, 404 and 379 in *L1710*, *L1817*, *L1730*, *O*. *sativa* and *O*. *longistaminata*, respectively) were expressed in the five lines. The relative expression level of miRNAs was detected from the frequency of their read counts using a deep-sequencing means. In the present study, the relative expression level of miRNAs varied from 1 to 856,496 reads among the five libraries (S8 Table). The expression levels of approximately 66% to 69% of the miRNAs were less than 100 reads in five lines, and the expression levels of 11–14% of the miRNA ranged from 100 to 500 reads, but the expression level of only 3% to 6% of the miRNAs surpassed 10,000 reads. Among these miRNAs, the expression levels of 11 miRNAs (osa-miR168a-5p, osa-miR528-5p, osa-miR166a-3p, osa-miR172d-3p, osa-miR166d-3p, osa-miR166f, osa-miR166b-3p, osa-miR166c-3p, osa-miR166j-3p, osa-miR166g-3p and osa-miR166h-3p) were more than 10,000 reads in five lines. The hierarchical clustering of all expressed miRNAs was performed to group five lines. We observed *L1817* and *O*. *sativa* formed a single group, and clustered with *L1710* and succeed to *L1730* (S4a Fig). Based on the results of Venn diagrams, we analyzed the commonly and specifically expressed miRNAs in five lines (S4b Fig). Similar to mRNA analysis, 291 expressed miRNAs were shared by all five lines, accounting for 56.73% of the 513 miRNAs. These co-expressed miRNAs demonstrated conservation between progeny lines and their parents. The uniquely expressed miRNAs in each line only accounted for a small fraction of the miRNAs, showing the most abundance in *O*. *longistaminata* (21), subsequently followed by *L1730* (19), *L1710* (10), *O*. *sativa* (8) and *L1817* (7).

### Differentially expressed miRNAs between progeny lines and their parents

**Fig 7:**
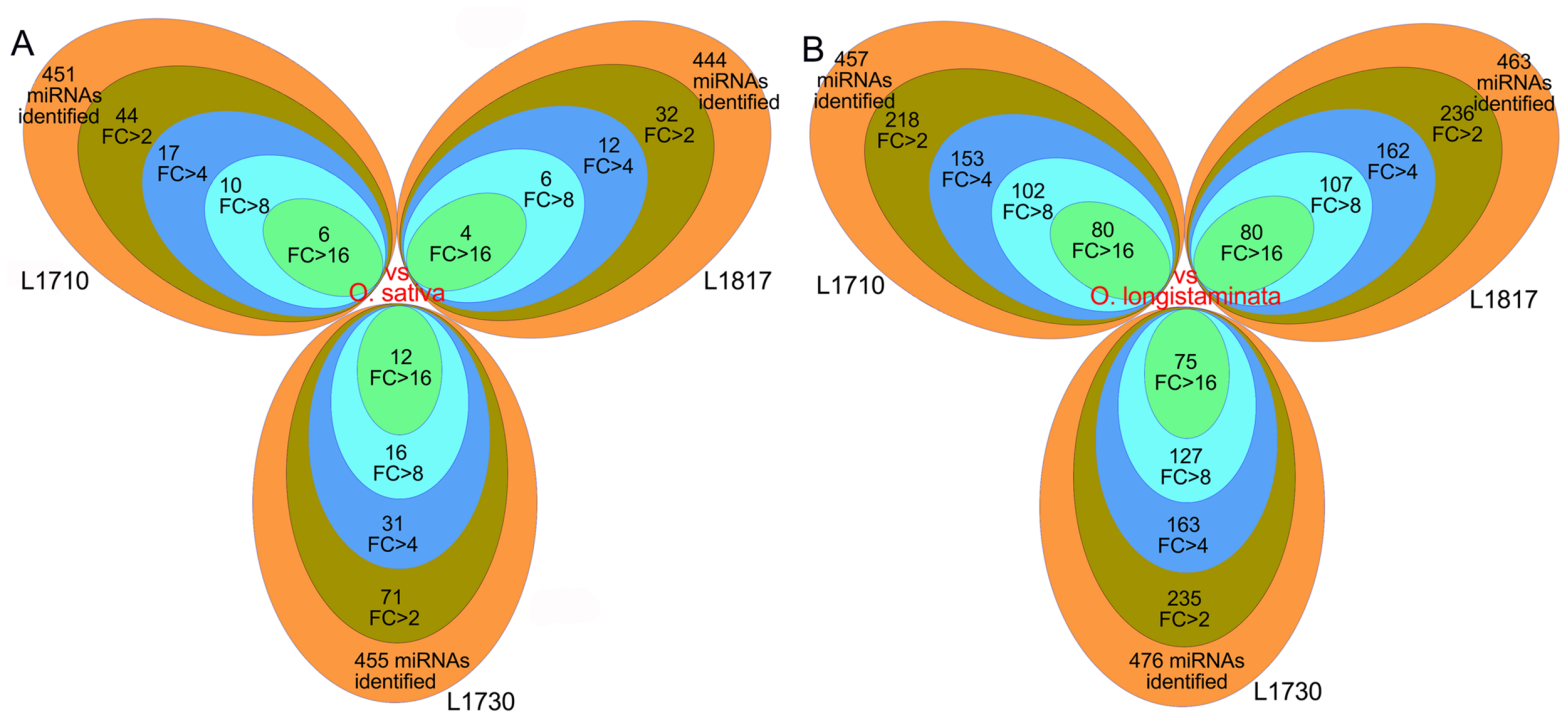
The number of differentially expressed miRNAs in the three progeny lines. FC, standing for fold change.

To explore the miRNAs expression patterns between progeny lines and their parents, each miRNA read count was normalized to transcripts per million (TPM). The number of differentially expressed miRNAs (log_2_Ratio≥1, P≤0.05) in progeny line/*O*. *longistaminata* comparison groups (more than 200) was much higher than that in progeny line/*O*. *sativa* comparison groups (less than 71) (Fig 7). The number of differentially expressed miRNAs in the *L1710*/*L1730* comparison group was higher than that in the *L1710*/*L1817* and the *L1817*/*L1730* comparison groups (Table 2). This finding was consistent with the plant phenotypic changes, and more differentially expressed miRNAs were observed in the comparison group with greater phenotype differences. The proportion of down-regulated miRNAs were comparable to the up-regulated miRNAs in three progeny line/*O*. *sativa* comparison groups, while 67% of the differentially expressed miRNAs were down-regulated in three progeny lines compared with *O*. *longistaminata* (Table 2). Approximately 67% of the miRNAs were down-regulated in *L1730* and *L1817* compared with *L1710*, and approximately 60% of the miRNAs were up-regulated in *L1730* compared with *L1817*. The differentially expressed miRNAs accounted for 47.70% to 50.97% of all expressed miRNAs in three progeny lines compared with *O*. *longistaminata*, but only accounted for 7.21% to 15.60% compared with *O*. *sativa* (Fig 7). Additionally, we calculated the differentially expressed miRNAs with a fold-change ≥16, accounting for 0.90% to 2.64% in progeny line/*O*. *sativa* comparison groups, but accounting for 15.76% to 17.51% in the progeny line/*O*. *longistaminata* comparison groups. These results suggested that both the number and fold-change of the differentially expressed miRNAs in progeny line/*O*. *longistaminata* comparison groups were higher than in the progeny line/*O*. *sativa* comparison groups. The proportion of differentially expressed miRNA among three progeny lines exhibited similarity, similar to the gene expression patterns, and this miRNA expression tendency may also be influenced by genome dosage.

**Table 2:** Differentially expressed miRNAs and their target genes in three progeny lines.

Comparison groups	Number of up-regulated miRNA	Number of down-regulated miRNA	Number of target genes of up-regulated miRNA	Number of target genes of down-regulated miRNA	Number of coherent target genes of up-regulated miRNA	Number of coherent target genes of down-regulated miRNAs
**A-vs-L1710**	27	17	772	454	1	4
**A-vs-L1817**	14	18	321	702	1	7
**A-vs-L1730**	35	36	549	1131	5	19
**B-vs-L1710**	74	144	1655	2550	60	112
**B-vs-L1817**	69	166	1584	2741	46	93
**B-vs-L1730**	76	159	1693	2626	53	110
**L1710-vs-L1817**	16	34	553	1069	6	4
**L1710-vs-L1730**	31	61	789	1412	22	3
**L1817-vs-L1730**	33	22	683	648	0	6

A and B stand for *O*. *sativa* and *O*. *longistaminata*, respectively.

For a more detailed analysis, we categorized differentially expressed miRNAs in three progeny lines into 5 major expression categories according to the gene parental expression level dominance analysis. Among the 12 expression patterns, more than 75% of the differentially expressed miRNAs showed parental ELD, similar to the observed gene expression patterns (S5 Fig). Differentially expressed miRNAs showing ELD-A (72% to 87%) significantly exceeded those showing ELD-B (5% to 9%) in each progeny line (S9 Table). In addition, the percentage of ELD-B miRNAs was clearly lower than their chromosome complements inherited from *O*. *longistaminata* in the three progeny lines. Furthermore, we observed that the number of down-regulated ELD-A miRNAs was higher than the up-regulated miRNAs, but an opposite trend was observed among the ELD-B miRNAs in the three progeny lines. In addition, similar to gene expression level dominance, the proportion of ELD-B miRNAs in *L1730* was higher than that in *L1817* and *L1710*.

### Integrated miRNA/mRNA expression analysis in the three progeny lines and their parents

Gene expression level is affected by a variety of factors, primarily controlled by the relative rates of transcription and RNA degradation, and miRNA post-transcriptional regulated mechanism is one of the gene expression regulatory factors. To further characterize the potential functions of miRNAs in the five lines, the target genes of miRNAs were predicted using two softwares (psRobot and Target Finder) with default parameters. Altogether, 4,791, 5,027, 5,011, 4,855 and 4,068 genes were predicted as target genes in *L1710*, *L1817*, *L1730*, *O*. *sativa* and *O*. *longistaminata*, respectively. Among these genes, 1,226 (*L1710*), 1,023 (*L1817*) and 1,680 (*L1730*) genes were predicted as target genes of differentially expressed miRNAs in progeny lines compared with *O*. *sativa*, and more than 4,000 genes were predicted as target genes in each progeny line/*O*. *longistaminata* comparison group (Table 2). More than 3,000 genes were identified as target genes of ELD-A miRNAs in each progeny line, with an average number of 16.56 target genes per miRNA, and nearly 500 genes were identified as target genes of ELD-B miRNAs, with an average number of 28.83 target genes per miRNA (S10 Table).

**Fig 8:**
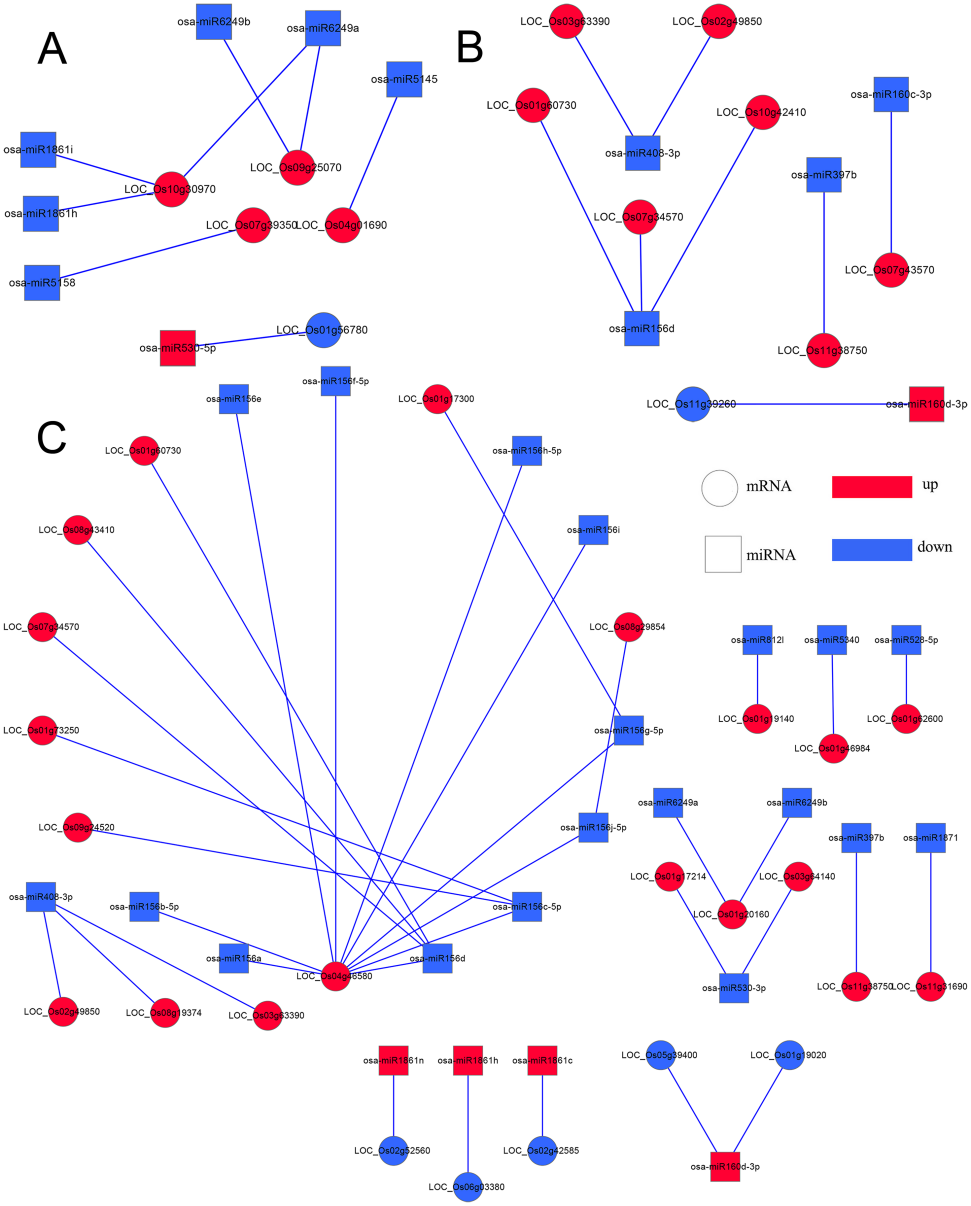
Integrated networks of differentially expressed miRNAs and their coherent target genes in three progeny lines compared with *O*. *sativa*. (A), (B) and (C) represent in *L1710*, *L1817* and *L1730*. Round rectangle represent miRNAs; Ellipse represent coherent target genes; red represent up-regulated; blue represent down-regulated.

**Fig 9:**
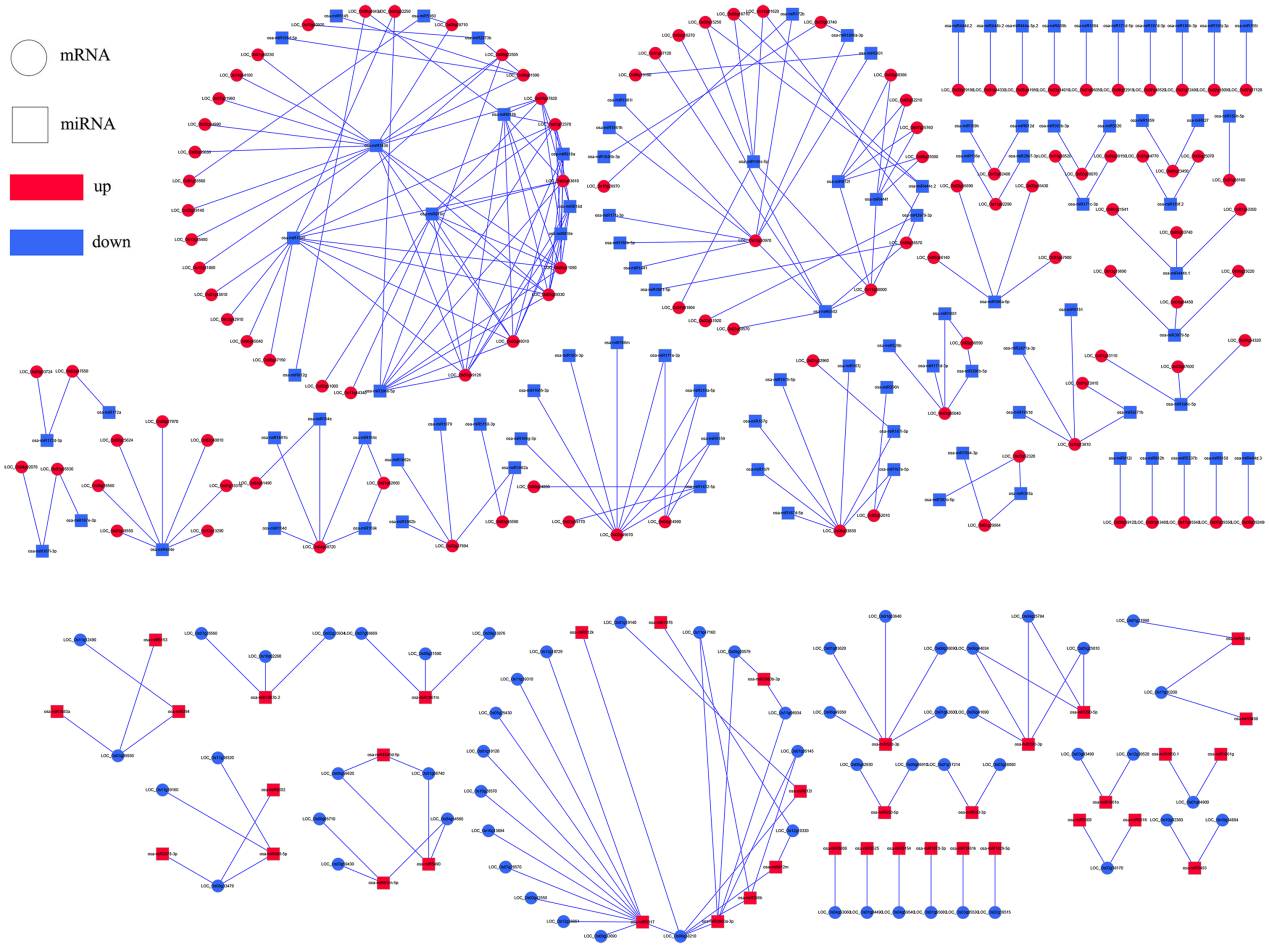
Integrated network of differentially expressed miRNAs and their coherent target genes in *L1710* compared with *O*. *longistaminata*. Round rectangle represent miRNAs; Ellipse represent coherent target genes; red represent up-regulated; blue represent down-regulated.

**Fig 10:**
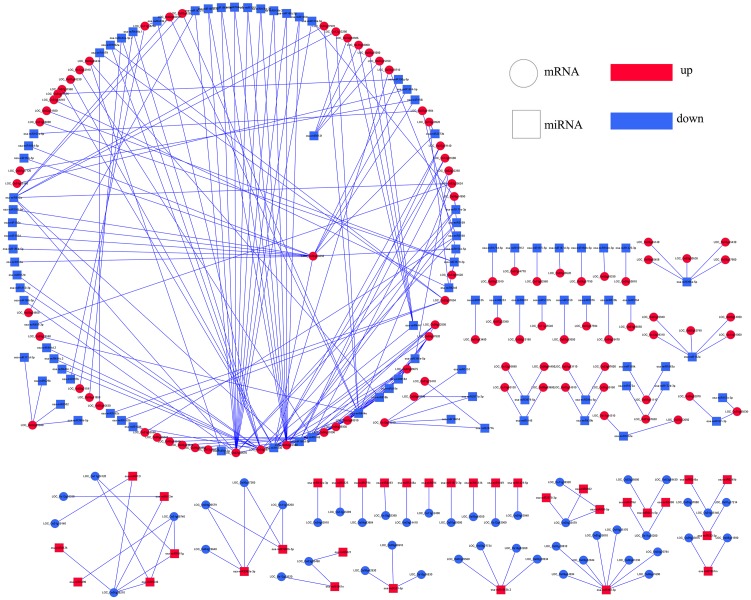
Integrated network of differentially expressed miRNAs and their coherent target genes in *L1817* compared with *O*. *longistaminata*. Round rectangle represent miRNAs; Ellipse represent coherent target genes; red represent up-regulated; blue represent down-regulated.

**Fig 11:**
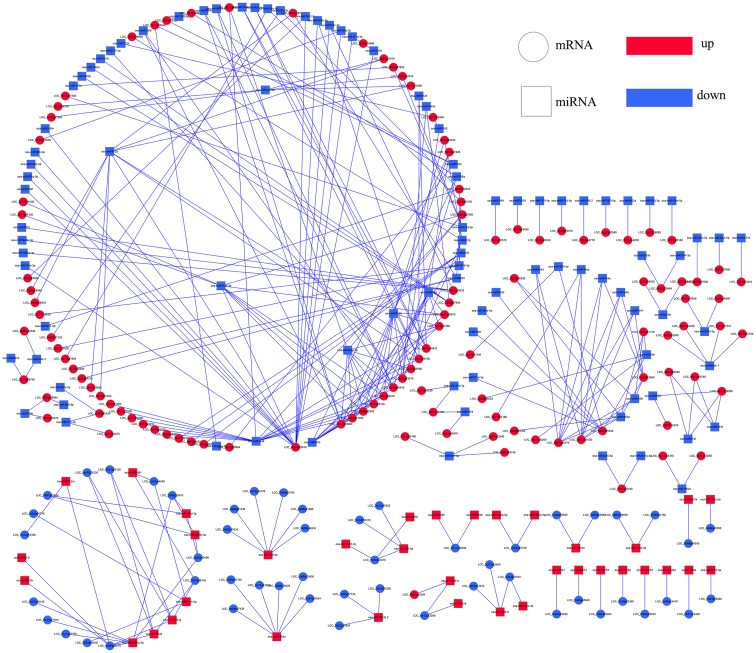
Integrated network of differentially expressed miRNAs and their coherent target genes in *L1730* compared with *O*. *longistaminata*. Round rectangle represent miRNAs; Ellipse represent coherent target genes; red represent up-regulated; blue represent down-regulated.

Among these target genes, we focused on coherent target genes inversely expressed with respect to miRNAs. One target gene of a miRNA was defined as a coherent target gene when its expression pattern was opposite that of the miRNA. For example, down-regulated genes were regarded as coherent target genes of up-regulated miRNAs, while other genes were defined as non-coherent target genes. Similarly, up-regulated genes were regarded as coherent genes related to the down-regulated miRNAs, and the remaining genes were considered non-coherent target genes. In the present study, 15.63%-63.76% of the differentially expressed miRNAs were predicted possessing coherent target genes in three progeny lines. In total, 5 (*L1710*), 8 (*L1817*) and 24 (*L1730*) target genes in three progeny line/*O*. *sativa* comparison groups, and 172 (*L1710*), 139 (*L1817*) and 163 (*L1730*) target genes in three progeny line/*O*. *longistaminata* comparison groups were regarded as coherent target genes (Table 2). Among those miRNAs, we observed the number of coherent target genes of down-regulated miRNAs was marked higher than up-regulated miRNAs in the three progeny lines (Figs 8–11). For example, the osa-miR1848, osa-miR396 and osa-miR818 families, corresponding to more than 10 coherent target genes in each progeny line, were down-regulated compared with *O*. *longistaminata*. The Osa-miR444 family possessing more than 20 coherent target genes, was down-regulated in *L1710* and *L1817* compared with *O*. *longistaminata*. The Osa-miR390 corresponding to more than 5 coherent target genes in each progeny line, was up-regulated compared with *O*. *longistaminata*. More than 50% of all the parental ELD miRNAs were observed with coherent target genes, and most of these miRNAs were ELD-A miRNAs and corresponded to less than 3 target genes (S6a–S6c Fig). Only 2 ELD-B miRNAs in *L1730* had coherent target genes (S6d Fig). Some miRNAs showed similar expression patterns among three progeny lines compared with their parents. For example, ELD-A miRNAs osa-miR3980a-3p was up-regulated and osa-miR1848 was down-regulated in the three progeny lines compared with *O*. *longistaminata*. To validate the correlation between the differentially expressed miRNAs and their coherent target genes, 6 miRNAs and their target genes were examined using the qRT-PCR method. The expression patterns between the 6 miRNAs and their coherent targets were basically inversed among five lines, which was consistent with the RNA-seq data (S7 Fig). These results suggest that the target gene prediction of the differentially expressed miRNAs was accurate.

**Fig 12:**
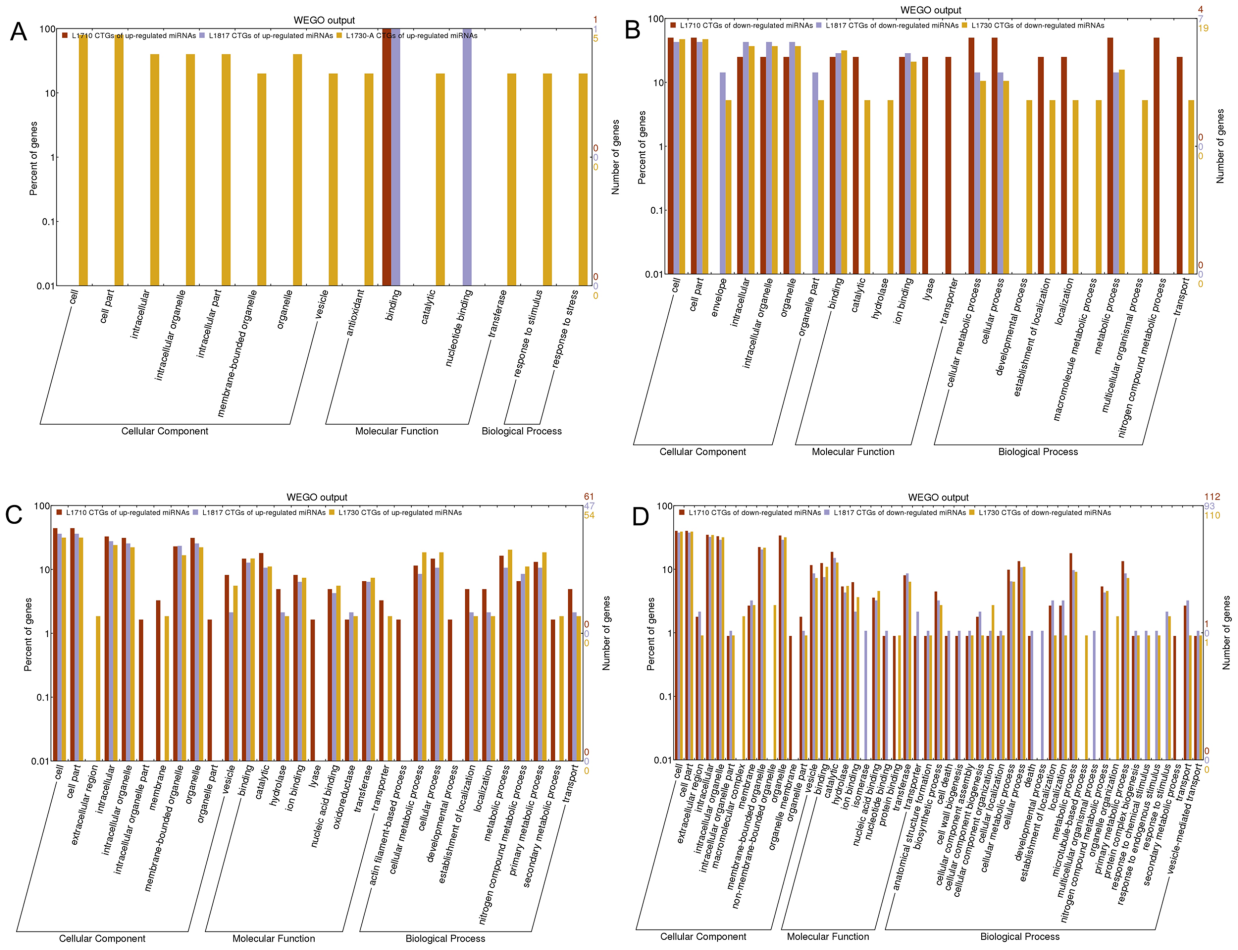
GO analysis of coherent target genes of differentially expressed miRNAs in the three progeny lines. Up-regulated (A) and down-regulated (B) miRNA in progeny lines compared with *O*. *sativa*. Up-regulated (C) and down-regulated (D) miRNA in progeny lines compared with *O*. *longistaminata*.

To determine the functional annotation of the differentially expressed miRNAs in each progeny line, GO analysis of miRNA coherent target genes was performed. In total, more GO terms involved in the target genes of differentially expressed miRNAs in three progeny line/*O*. *longistaminata* comparison groups were detected than in three progeny line/*O*. *sativa* comparison groups. In addition, the target genes of down-regulated miRNAs were assigned to more GO terms than that of up-regulated miRNAs in the three progeny lines. (Fig 12). The target genes of down-regulated miRNAs among three progeny lines compared with *O*. *sativa* assigned to 10 terms in three progeny lines, 6 terms in two progeny lines, and 7 terms in one progeny line. Three terms (‘lyase’, ‘transporter’ and ‘nitrogen compound metabolic process’) and 4 terms (‘hydrolase activity’, ‘developmental process’, ‘macromolecule metabolic process’ and ‘multicellular organismal process’) were only assigned by the target genes of down-regulated miRNAs in *L1710* and *L1730*, respectively. Among the three progeny lines/*O*. *longistaminata* comparison groups, target genes of down-regulated miRNAs and up-regulated miRNAs were primarily enriched in 22 and 33 terms, respectively (Fig 12c and 12d). Some terms, such as ‘intracellular organelle’, ‘membrane-bounded organelle’, ‘oil binding’, ‘nucleic acid binding’, ‘oxidoreductase activity’, ‘transferase’ ‘cellular metabolic process’ and ‘primary metabolic process’, were enriched by not only target genes of up-regulated miRNAs, but also by that of down-regulated miRNAs in the three progeny lines. Most target genes participating in these terms corresponded to multiple miRNAs, indicating that these terms might be regulated by multiple miRNAs. For example, the NAC gene (*OMTN4*, LOC_Os06g46270) participated in ‘nucleic acid binding’ was up-regulated in *L1730* compared with *O*. *longistaminata*, and was the target gene of osa-miR169k, osa-miR1863a, osa-miR164 family. NAC transcription factor gene (*OsNAC2*, LOC_Os04g38720) involved in ‘cell part’ term was up-regulated in *L1710* and *L1730* compared with *O*. *longistaminata*, and was the target gene of osa-miR164c, osa-miR164d, osa-miR164e, osa-miR169k, osa-miR1863a and osa-miR1861b. *OsGH3-9* (LOC_Os07g38890) was the target gene of down-regulated osa-miR812g in the three progeny lines compared with *O*. *longistaminata*. In ‘hydrolase activity’, *OsGRF12* (LOC_Os04g48510) and *OsGRF7* (LOC_Os12g29980) up-regulated in *L1730* were predicted to be target genes of osa-miR396 family, osa-miR5150-5p/3p and osa-miR818e. The *LOG* (LOC_Os01g40630) gene, involved in ‘cytokinin metabolic process’, was the coherent target gene of osa-miR1879 and was up-regulated in *L1817* compared with *O*. *longistaminata*. The target genes of those down-regulated miRNAs were involved in more GO terms than that of up-regulated miRNAs, indicating that down-regulated miRNAs might play more important roles in gene expression regulation in three progeny lines.

Owing to the small number of ELD-B miRNAs, no coherent target genes of ELD-B miRNAs were observed in *L1710* and *L1817*, and 5 coherent target genes of ELD-B miRNAs were observed in *L1730*. Hence, we only performed GO analysis of the coherent target genes of ELD-A miRNAs to figure out the potential function in the three progeny lines (S8 Fig). The results revealed that only the coherent target genes in *L1730* enriched in ‘macromolecular complex’ and ‘non-membrane-bounded organelle’, and the ELD-A genes number in these 2 terms in *L1730* significantly exceeded that in *L1710* and *L1817* (Fig 4). Moreover, the coherent target genes specifically enriched in ‘developmental process’, ‘multicellular organismal development’ and ‘multicellular organismal process’ in *L1817*. Coherent target genes were enriched in ‘response to stimulus’, ‘response to chemical stimulus’ and ‘response to endogenous stimulus’ in *L1817* and *L1730*. Three terms, ‘death’, ‘cell death’ and ‘cell wall biogenesis’, were enriched in coherent target genes in *L1710* and *L1817*, without *L1730*. The number of target genes enriched in ‘cellular component organization’ in *L1730* was much higher than that in *L1710* and *L1817*, suggesting that miRNAs with coherent target genes might play key roles in cell growth and metabolism in *L1730* during the rice jointing stage. These findings indicated that ELD-A miRNAs might regulate gene expression changes of cellular component category and biological process category.

### Validation of the RNA-Seq data using qRT-PCR

To assess the validity and reliability of sequencing data, and confirm the expression profiles of genes and miRNAs among the five libraries, 12 expressed genes (S9 Fig) and 6 expressed miRNAs (S10 Fig) were randomly selected to examine the RNA-Seq analysis using qRT-PCR. The qRT-PCR results of genes and miRNAs were basically consistent with that of the RNA-Seq data, indicating that the sequencing data produced in the present study were reliable and could be subjected to further analysis.

## Discussion

Using progeny lines of a backcross introgression line and their parental species as models to explore gene expression divergence is a novel means to address the mechanism of gene introgression affecting progeny phenotypic variations. The transcriptome and small RNA data generated in the present study were useful to understand the variations in gene expression between the progeny and their parents, and identify the complex molecular mechanism of plant height diversity in three progeny lines, which will provide additional insight into the mechanism underlying rice hybrid and backcross processes.

### Expression of genes and miRNAs was primarily influenced by the genome dosage of recurrent parent in backcrossed progenies

Plant hybrid is a combination of divergent genomes, leading to instant and profound genome modifications in various ways, including both structural and epigenetic mechanisms [38], and subsequently leading to expression changes of gene [39, 40]. Many studies have been performed to decipher the potenial molecular basis for hybrids, allopolyploids and NILs through detailed annotation of the transcriptome and detection of the miRNA regulation mechanism in progenies and their parents [11, 12, 21, 39–41]. In hybrid rice *LYP9*, the transcriptome profiles of super-hybrid rice *LYP9* were closer to the female parent at the early stages of development but similar to the male parent at later stages, consistent with their morphological characteristics [3]. In the present study, both the gene and miRNA expression patterns were similar to *O*. *sativa* in progeny lines, but their phenotypes were not all closer to *O*. *sativa*. The gene and miRNA expression profiles in *L1710* and *L1817* were closer to *O*. *sativa* than that in *L1730*. Additionally, gene and miRNA expression profiles in *L1730* were closer to *O*. *longistaminata*, which was consistent with that the plant height of *L1730* is closer to the *O*. *longistaminata* compared with the other lines. In previous studies, the number of co-expressed genes or miRNAs was higher than specifically expressed genes or miRNAs between progeny and its parents [15, 28]. However, the number of specifically expressed genes was higher than the co-expressed genes in super-hybrid rice *LY2108* and its parents [42]. In the present study, the numbers of co-expressed genes and miRNAs were far more than that of specifically expressed genes and miRNAs in five lines. Additionally, more co-expressed genes and miRNAs existed between progeny line and *O*. *sativa* than that between progeny line and *O*. *longistaminata*. Furthermore, the number of specifically expressed genes and miRNAs in *O*. *longistaminata* were highest among the five lines, and followed by *L1730*. These co-expressed genes and miRNAs probably related to the basic growth that contained conservative function in rice jointing stage, while specifically expressed genes and miRNAs, probably contribute to the special growth and development processes in the five lines, particularly in *L1730*.

In previous studies, the maternal genome was demonstrated to have an advantage over the paternal genome, and the expression of DEGs and differentially expressed miRNAs were closely associated with the parental genome dosage in progeny [15, 21, 37]. In seedlings and spikes of synthetic hexaploid wheat and spikes of natural allohexaploids wheat, the genes and miRNAs showed expression-level dominance to the maternal parent (AB), but showing expression-level dominance to the maternal parent (D) in synthetic hexaploid wheat seeds [21]. However, the expression of triploid hybrid rice is closer to the paternal parent for the paternal subgenome (BC) comprised of two genomes and the maternal subgenome (A) was comprised of one genome [40]. In the present study, the obvious diversity of genes and miRNAs showed parental ELD patterns, indicating that genes and miRNAs of one parent (primarily *O*. *sativa*) were dominantly expressed in three progeny lines. These findings were consistent with previously reported about an inter-specific hybrid rice [40] and indicated that the *O*. *sativa* genome was globally dominant over the *O*. *longistaminata* genome due to dosage advantage after hybrid and backcross process. Together, this analysis further showed that, the expression of genes and miRNAs showed parental expression level dominance in progeny of BILs rice. More differentially expressed genes and miRNAs were observed in *Brassica* hexaploid (BBCCAA) compared with male parent *B*. *rapa* (AA) than that compared with female parent *B*. *carinata* (BBCC), which may be affected by its genome dosage [15, 28]. In the present study, more differentially expressed genes and miRNAs were observed between progeny and *O*. *longistaminata*, for most of the chromosome complements of progeny were inherited from *O*. *sativa*. These results suggested that the expression of genes and miRNAs in progeny was influenced by the parental genome dosage of the progeny, particular the differentially expressed genes and miRNAs.

The DEGs participated in complex pathways, such as ‘carbohydrate metabolism’, ‘plant hormone signal transduction’ and ‘photosynthesis-related pathways’, were associated with phenotypic variation in hybrid progeny [21, 40, 41]. In nascent hexaploid wheat, the expression of ‘development’ related genes are similar to the female parent and that of ‘adaptation’ related genes display similar to male parent in the progeny, which may be conducive to stress responses and photoperiod adaptability [21]. Furthermore, we noticed that DEG_UHP_ were enriched in ‘cellular metabolic process’, ‘metabolic process’ and ‘primary metabolic process’ in all progeny lines, and these three terms were statistically significant differences among three progeny lines. The coherent target genes of differentially expressed miRNAs were primarily enriched in ‘cellular metabolic process and ‘primary metabolic process’ in three progeny lines compared with *O*. *longistaminata*. Thus, ‘cellular metabolic process’ and ‘primary metabolic process’ were differently expressed among three progeny lines. Nevertheless, genes displayed ELD toward *O*. *sativa* and the coherent target genes of miRNAs that displayed ELD toward *O*. *sativa* were significant enriched in ‘cellular metabolic process’ and ‘primary metabolic process’ in three progeny lines (Fig 6b, S8 Fig). Therefore, we speculated the primary metabolism and cellular metabolism related genes and miRNAs were primarily influenced by the genome dosage of *O*. *sativa* and might be contributed to the internode elongation and plant height variations among three progeny lines.

### Regulation of genes and miRNAs related with plant hormones and cell wall might contribute to the height variations in backcrossed progeny

To achieve high-quality rice, both high yielding potential and optimal architecture, determined by plant height to a large extent, were required. Rice height is primarily determined according to rice stems, in which internode number and internode length play critical roles. Internode length is determined by cell division in the meristem and cell elongation in the elongated region, which is influenced by plant hormones and the cell wall components biosynthesis and metabolism [43–48]. The plant hormones, such as auxin, gibberellins (GA), cytokinin, jasmonic acid (JA) and brassinosteroid (BR), are important regulators that determine plant height by regulating internode elongation [43, 49–51]. The increased expression of auxin-responsive genes (*GH3*) inhibited the plant growth in cotton and result in dwarf plant [19]. Among these data, the expression of *GH3* was up-regulated in *L1710* and *L1817* compared with *O*. *longistaminata*, but showed little change in *L1730* compared with *O*. *longistaminata*. Additionally, *GH3* was down-regulated in *L1730* compared with *O*. *sativa*. PIN proteins as auxin output vector mediate auxin flow and the expression of *PIN* genes are influenced by exogenous auxin and other hormones [52]. OsPIN10b was monocot-specific PIN protein, and the expression of *OsPIN10b* was up-regulated in *L1730* compared with *L1817* and *L1710* in the present study. BR biosynthesis-deficient mutants showed dwarf phenotypes with reduced cell elongation, shorter leaf sheaths and erect dark green leaves [44]. In the KEGG analysis, the ‘BR biosynthesis pathway’ was up-regulated in *L1710* and *L1817*, but down-regulated in *L1730*, and ‘diterpenoid biosynthesis’ was up-regulated in progeny lines, except in the *L1730*/*O*. *sativa* comparison group. *OsNAC2* directly participated in the GA pathway, delaying flowering time and reducing the length of the internodes in rice [53]. The expression of *OsNAC2* was up-regulated and regulated by 6 miRNAs (osa-miR164c, osa-miR164d, osa-miR164e, osa-miR169k, osa-miR1863a and osa-miR1861b) in *L1710*, indicating that these miRNAs might participate in the regulation of internodes elongation. *CYP714B1* encodes GA 13-oxidase and reduces the activity of GAs, thereby influencing the length of rice internodes [54]. In the present study, *CYP714B1* was up-regulated in *L1817* and *L1730*, indicating that its expression might contribute to the internode elongation of the three progeny lines. Moreover, some genes were coordinately regulated through GA and BR in rice, such as *OsGSR1 and D62* [50, 55]. The *OsGSR1*, a positive regulate factor in GA signaling, acts key role in the interactions between BR and GA signaling pathways in rice [55]. In the BR-insensitive dwarf mutant, the BR-responsive gene (*D62*) affected the GA metabolism and mediated the interaction between GA and BR in rice [56]. Among these data, the expression of *D62* and *OsGSR1* were up-regulated in the three progeny lines, suggesting that the interaction between BR and GA signaling might be enhanced after hybridization and backcrossing process. These results indicated that the expression of genes and miRNAs related to BR biosynthesis and GA signaling pathways might contribute to hormone regulation and changes in plant height in the three progeny lines. Furthermore, *LOG* participates in bioactive cytokinin synthesis through encoding a cytokinin-activating enzyme [51]. Many studies have shown that miRNAs play important roles through regulating the target genes in the plants growth [16–18, 21, 27]. The study of Wen et al. provided important resources for the investigation of miRNA functions in rice domestication [18]. Among these data, *LOG* was involved in 2 GO terms, ‘intracellular membrane-bounded organelle’ and ‘cytokinin metabolic process’. In addition, *LOG* was the coherent target gene of osa-miR1879, and the expression level of osa-miR1879 in three progeny lines was dominance toward *O*. *sativa* but lower than that in *O*. *longistaminata*. Therefore, we speculated that osa-miR1879 might be a regulator in the regulation of ‘cytokinin metabolic process’ at the jointing stage of three progeny lines. In short, complex expression changes of these hormones related genes might contribute to the height variations in the three progeny lines after hybridization and backcrossing, and it will be interesting to explore more miRNAs who play a role as regulator in plant hormone signal transduction.

During plant growth and development, the cell wall plays an important role in plant architectural design, including influencing cell shape and mechanical strength. The plant cell wall is primarily comprised 3 polysaccharides, including cellulose, hemicellulose and pectin, and the biosynthesis of these polysaccharides is determined by the CESA (cellulose synthase active subunit) super-family and the CSL (cellulose synthase-like) super-family [48, 57]. Three genes (*OsCESA4*, *OsCESA7* and *OsCESA9*) involving cellulose synthase subunit participated in the biosynthesis of secondary cell wall, and played considerable roles in plant growth [43, 58]. The expression of three *OsCESA* genes displayed ELD toward *O*. *sativa* and were highest in *O*. *longistaminata*, consistent with changes of the third internode cell length in the five lines, indicating that *OsCESA* genes might contribute to the various cell lengths of the three progeny lines. *OsCSLD4* (LOC_Os12g36890) affects the cell-wall formation and plant growth, whose deficiency can lead to a significantly reduced plant height and the apparent structural defects in rice primary walls [59]. In the present study, the expression level of *OsCSLD4* was highest in *L1730*, and lowest in *L1710*. Guevara et al. observed that OsUGE1 might play an important role in the distribution of carbohydrate by altering the contents of sucrose, cellulose, galactose and glucose in rice cell walls [60]. In the present study, *OsUGE1* involved in ‘vesicle’ and ‘primary metabolic process’ likely participated in the biosynthesis of the internode cell walls in the three progeny lines. These results indicated that some cell wall related genes might play important roles in plant height by influencing cell division and formation. Through regulating the *OsSPL14* (LOC_Os08g39890) gene osa-miR156 improved the grain yield of rice through changing its plant architecture [22]. In the present study, osa-miR156 was down-regulated in the three progeny lines compared with *O*. *longistaminata*, and the expression levels of *OsSPL14* in the three progeny lines were higher than that in *O*. *longistaminata*. *Os-GRF1* has been reported as potential regulator of stem elongation in rice [46]. In *Arabidopsis*, *OsGRF* genes participated in the cell division and differentiation during leaf development and were targeted by miR396 [61]. In the present study, up-regulated *OsGRF12* and *OsGRF7* were regulated through down-regulated osa-miR396 family, osa-miR5150-5p/3p and osa-miR818e in three progeny lines compared with *O*. *longistaminata*. Therefore, we predicted that miRNAs might participate in internode cell division and stem elongation through regulating the target genes that contributed to the variations of plant height in three progeny lines during rice jointing stage. Cell elongation is not only correlated with the biosynthesis of cellulose, hemicellulose and pectin, but is also influenced by cell-wall loosening, which is regulated by cell-wall loosening factors. Expansin was involved in cell expansion through regulating cell-wall loosening activity during cell-wall modification [62]. In the present study, expansin gene (*OsEXP1*) was up-regulated in *L1817* compared with *O*. *sativa*, but down-regulated in *L1730* compared with *O*. *longistaminata*. The construction of xyloglucan cross-links is influenced by xyloglucan endotransglucosylases/hydrolases (XTHs), XTH-related gene *OsXTH8* is potentially related to cell elongation and affects the height of mutants [63]. In the present study, the expression of *OsXTH8* was up-regulated in *L1710* and *L1817* compared with *O*. *longistaminata*, but down-regulated in *L1817* and *L1730* compared with *O*. *sativa*. Glucanases have been recognized as cell-wall loosening enzymes through hydrolyzing the major parts of the plant cell-wall (1,3;1,4)- β- D -glucans [64]. Plant β-1,3-glucanases are participated in the defense and development of plant, and the silence of *Osg1* results in delayed development and reduced plant height in a *japonica* rice variety [65]. In this study, *Osg1* involving in the ‘metabolic process’ was up-regulated in *L1730* compared with *L1817* and *L1710*. According to these results, we predicted that the genes and miRNAs related to plant hormones and cell wall might play important roles in cell division and elongation, contributing to the plant height of the three progeny lines at the jointing stage.

In summary, the transcription and sRNAs analysis utilizing HiSeq technology provided an integrative method to investigate molecular mechanism of hybridization and backcrossing. The present study produced sufficient and reliable data for the exploration of gene and miRNA expression variations in the three progeny lines of backcrossed introgression line (BC_2_F_12_). Most genes and miRNAs expression patterns were toward recurrent parent in the three progeny lines, which might be influenced by their genome components. In the present study, GO and KEGG pathway analysis revealed some genes involved in specific biological processes and pathways, which might contribute to the plant height changes of the three progeny lines. The genes participating in the synthesis of plant hormones and hormone signal transduction and the genes related to cell wall might act crucial roles in the plant height of the three progeny lines at the jointing stage. This information from the present study will enhance the current understanding of the molecular mechanisms of interspecific hybrid backcrossing and may be useful for further analysis of the molecular regulation in the variations of plant height among the three progeny lines.

## Supporting information

S1 Fig The composition of parental chromosome complements in three progeny lines. The blue color standing for chromosome complements inherited from *O*. *longistaminata*; white color standing for chromosome complements inherited from *O*. *sativa*; green color standing for heterozygosity chromosome complements inherited from *O*. *longistaminata* and *O*. *sativa*.
(TIF)

S2 Fig GO analysis between ELD-A genes and ELD-B genes in three progeny lines. The x-axis represents the name of the GO subcategories. The right y-axis indicates the number of genes expressed in a given sub-category. The left y-axis indicates log (10) scale, the percent of a specific category of genes in that main category. GO terms showed statistically significant differences (P value<0.05) were denoted by stars. A and B stand for *O*. *sativa* and *O*. *longistaminata*, respectively.
(TIF)

S3 Fig The length distribution of small RNAs in the five libraries. (TIF)

S4 Fig Hierarchical cluster analysis of expressed miRNAs and co-expressed miRNAs in three progeny lines and their parents. (A) Hierarchical cluster analysis of expressed miRNAs. (B) Co-expressed and specially expressed miRNAs. The branch length indicates the degree of variance, and the color represents the logarithmic intensity of expressed miRNAs. Species groups are shown as columns, and individual expressed miRNAs are arrayed in rows.
(TIF)

S5 Fig Twelve patterns of miRNAs in three progeny lines. A and B stand for *O*. *sativa* and *O*. *longistaminata*, respectively. ELD-A miRNA indicated miRNA expression level is similar to *O*. *sativa*, but is differential compared with *O*. *longistaminata*; ELD-B miRNA indicated miRNA expression level is similar to *O*. *longistaminata*, but is differential compared with *O*. *sativa*.
(TIF)

S6 Fig Integrated networks of parental ELD miRNAs and their coherent target genes in the three progeny lines. (A), (B) and (C) Integrated ELD-A miRNAs and their coherent target genes network in *L1710*, *L1817* and *L1730*; (D) Integrated ELD-B miRNAs and their coherent target genes network in *L1730*. A and B stand for *O*. *sativa* and *O*. *longistaminata*, respectively; Round rectangle represent miRNAs; Ellipse represent coherent target genes; red represent up-regulated; blue represent down-regulated.
(TIF)

S7 Fig Validation of differentially expressed miRNAs and their potential coherent target genes via quantitative qRT-PCR. *Actin1* and U6 snRNA are used as internal reference gene and small RNA, respectively.
(TIF)

S8 Fig GO analysis of coherent target genes of ELD-A miRNAs among three progeny lines. A stand for *O*. *sativa*.
(TIF)

S9 Fig Validation via quantitative qRT-PCR of DEGs obtained from deep sequencing. *Actin1* is used as internal reference gene. The data of quantitative RT-PCR validation are expressed as the mean SD after normalization. Error bars indicate the standard deviation (±SD) of three replicates. Blue polylines are derived from RNA-Sqe data.
(TIF)

S10 Fig Validation via quantitative qRT-PCR of differentially expressed miRNAs obtained from deep sequencing. U6 snRNA is used as a reference small RNA. The data of real-time qPCR validation are expressed as the mean SD after normalization. Error bars indicate the standard deviation (±SD) of three replicates. Blue polylines are derived from RNA-Seq data.
(TIF)

S1 Table The primers designed for real-time quantitative PCR reaction. (XLSX)

S2 Table The primers designed for reverse-transcription and real-time quantitative PCR reaction. (XLSX)

S3 Table Summary of mRNA sequencing read in five libraries. (DOCX)

S4 Table Distribution of mRNA sequence length in five libraries. (DOCX)

S5 Table Numbers of considerably changed KEGG pathways in three progeny lines. (DOCX)

S6 Table The percentage of five categories in three progeny lines mRNA analysis. (DOCX)

S7 Table Distribution of small RNAs classes in five lines. (DOCX)

S8 Table Distribution of abundance of miRNAs in three progeny lines and their parents. (DOCX)

S9 Table The percentage of five categories in three progeny lines miRNA analysis. (DOCX)

S10 Table Number of target gene of ELD miRNA in the three progeny lines. (XLSX)
