# Supplementary figures and images for: Integrated analysis of mRNA and miRNA expression profiling in rice backcrossed progenies (BC2F12) with different plant height
Source: PLoS One. 2017 Aug 31;12(8):e0184106. doi: 10.1371/journal.pone.0184106 (PMC5578646; doi:10.1371/journal.pone.0184106)

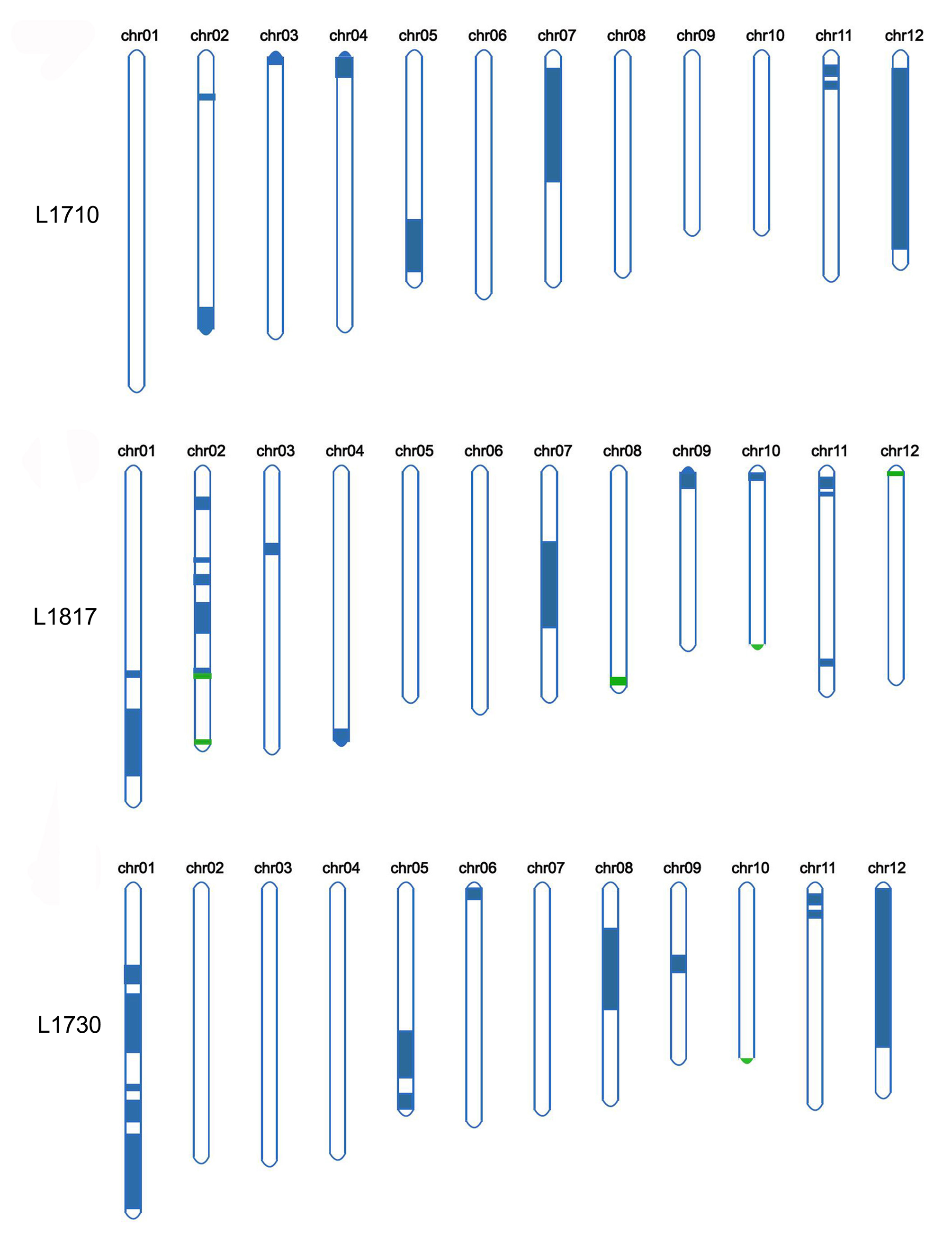

Supplement: S1 Fig — The blue color standing for chromosome complements inherited from O. longistaminata; white color standing for chromosome complements inherited from O. sativa; green color standing for heterozygosity chromosome complements inherited from O. longistaminata and O. sativa. (TIF) [file pone.0184106.s001.tif]

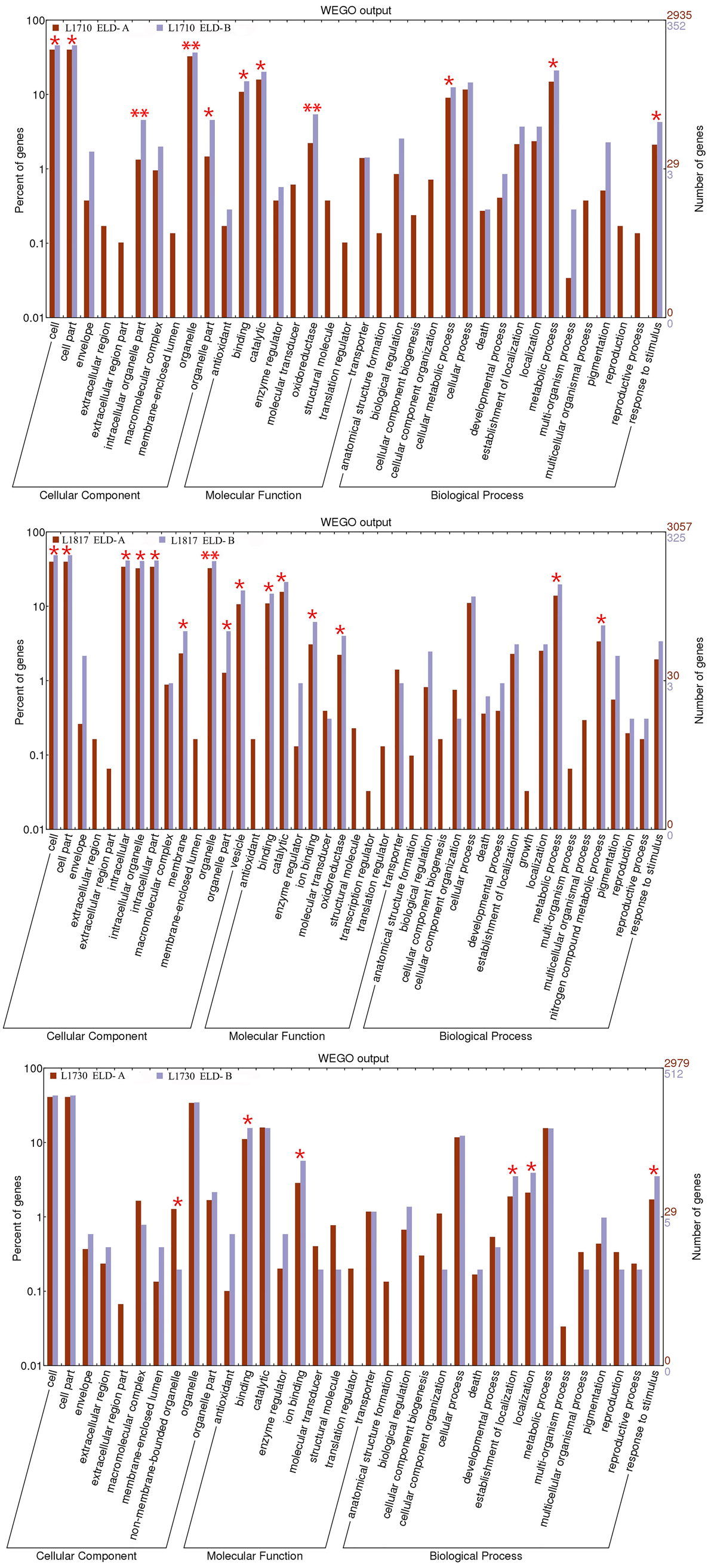

Supplement: S2 Fig — The x-axis represents the name of the GO subcategories. The right y-axis indicates the number of genes expressed in a given sub-category. The left y-axis indicates log (10) scale, the percent of a specific category of genes in that main category. GO terms showed statistically significant differences (P value<0.05) were denoted by stars. A and B stand for O. sativa and O. longistaminata, respectively. (TIF) [file pone.0184106.s002.tif]

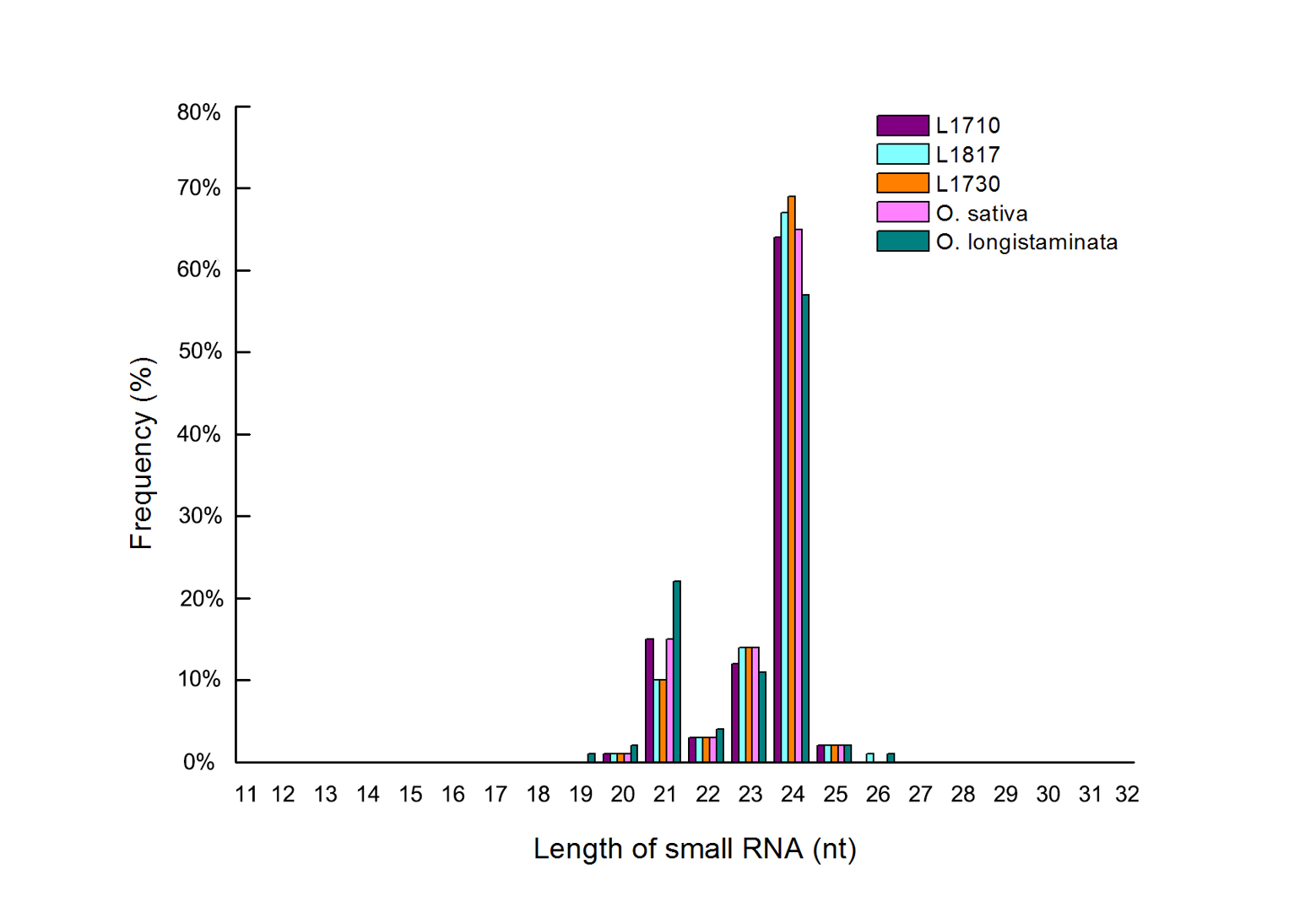

Supplement: S3 Fig — (TIF) [file pone.0184106.s003.tif]

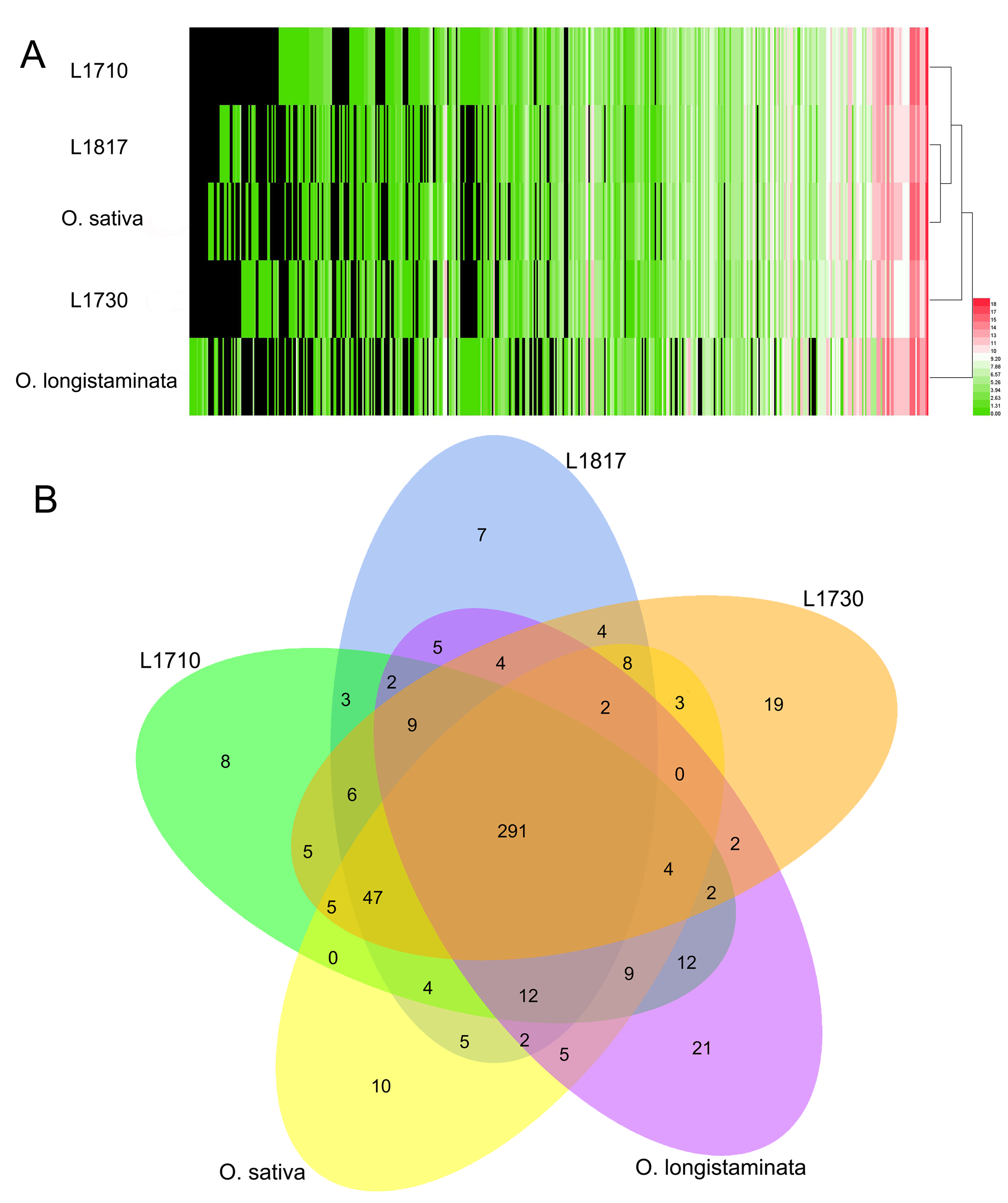

Supplement: S4 Fig — (A) Hierarchical cluster analysis of expressed miRNAs. (B) Co-expressed and specially expressed miRNAs. The branch length indicates the degree of variance, and the color represents the logarithmic intensity of expressed miRNAs. Species groups are shown as columns, and individual expressed miRNAs are arrayed in rows. (TIF) [file pone.0184106.s004.tif]

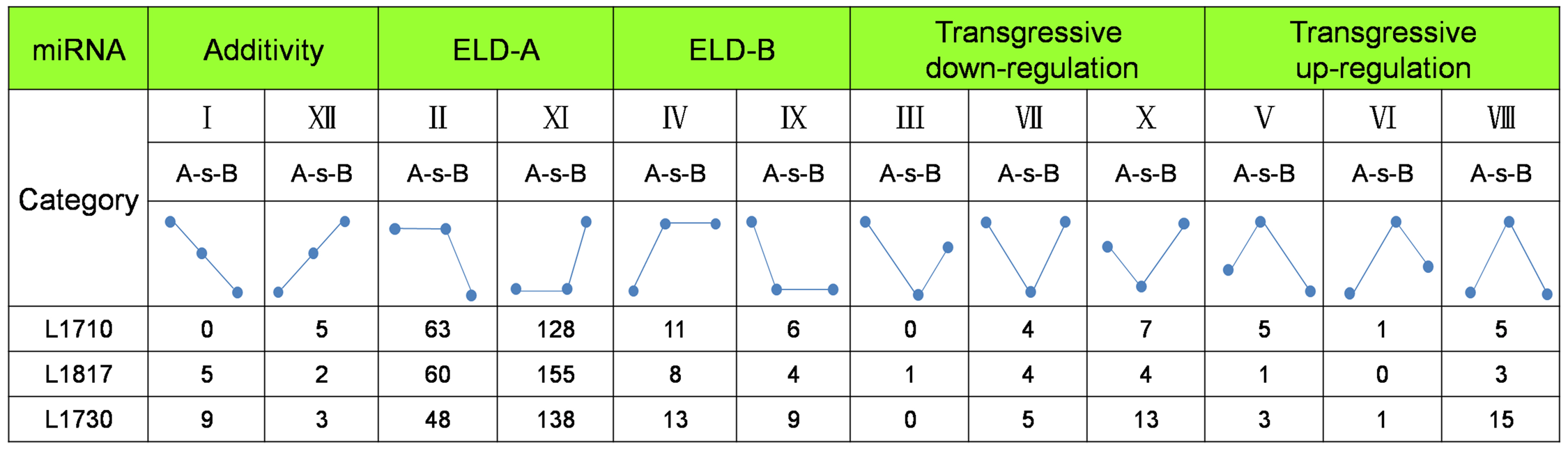

Supplement: S5 Fig — A and B stand for O. sativa and O. longistaminata, respectively. ELD-A miRNA indicated miRNA expression level is similar to O. sativa, but is differential compared with O. longistaminata; ELD-B miRNA indicated miRNA expression level is similar to O. longistaminata, but is differential compared with O. sativa. (TIF) [file pone.0184106.s005.tif]

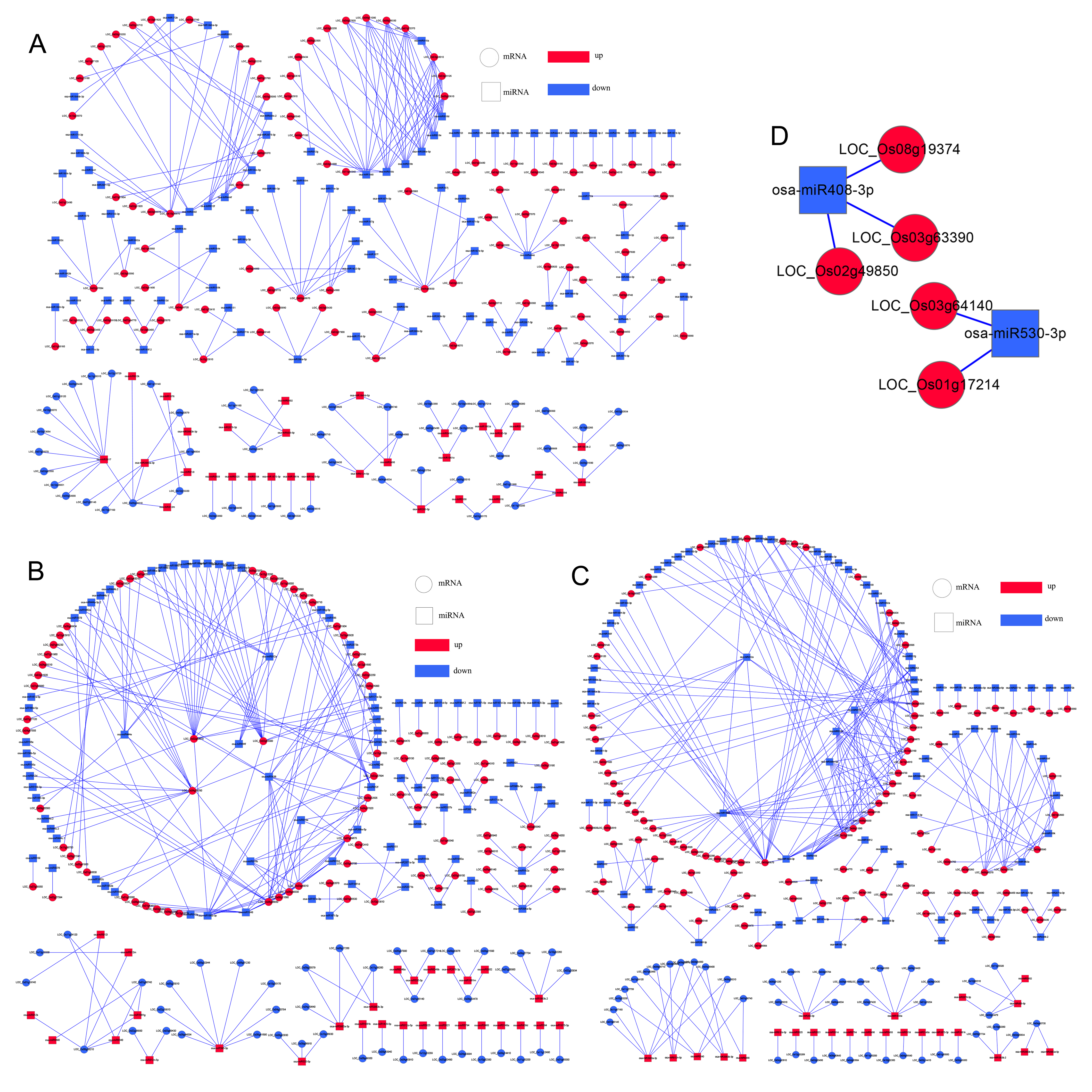

Supplement: S6 Fig — (A), (B) and (C) Integrated ELD-A miRNAs and their coherent target genes network in L1710, L1817 and L1730; (D) Integrated ELD-B miRNAs and their coherent target genes network in L1730. A and B stand for O. sativa and O. longistaminata, respectively; Round rectangle represent miRNAs; Ellipse represent coherent target genes; red represent up-regulated; blue represent down-regulated. (TIF) [file pone.0184106.s006.tif]

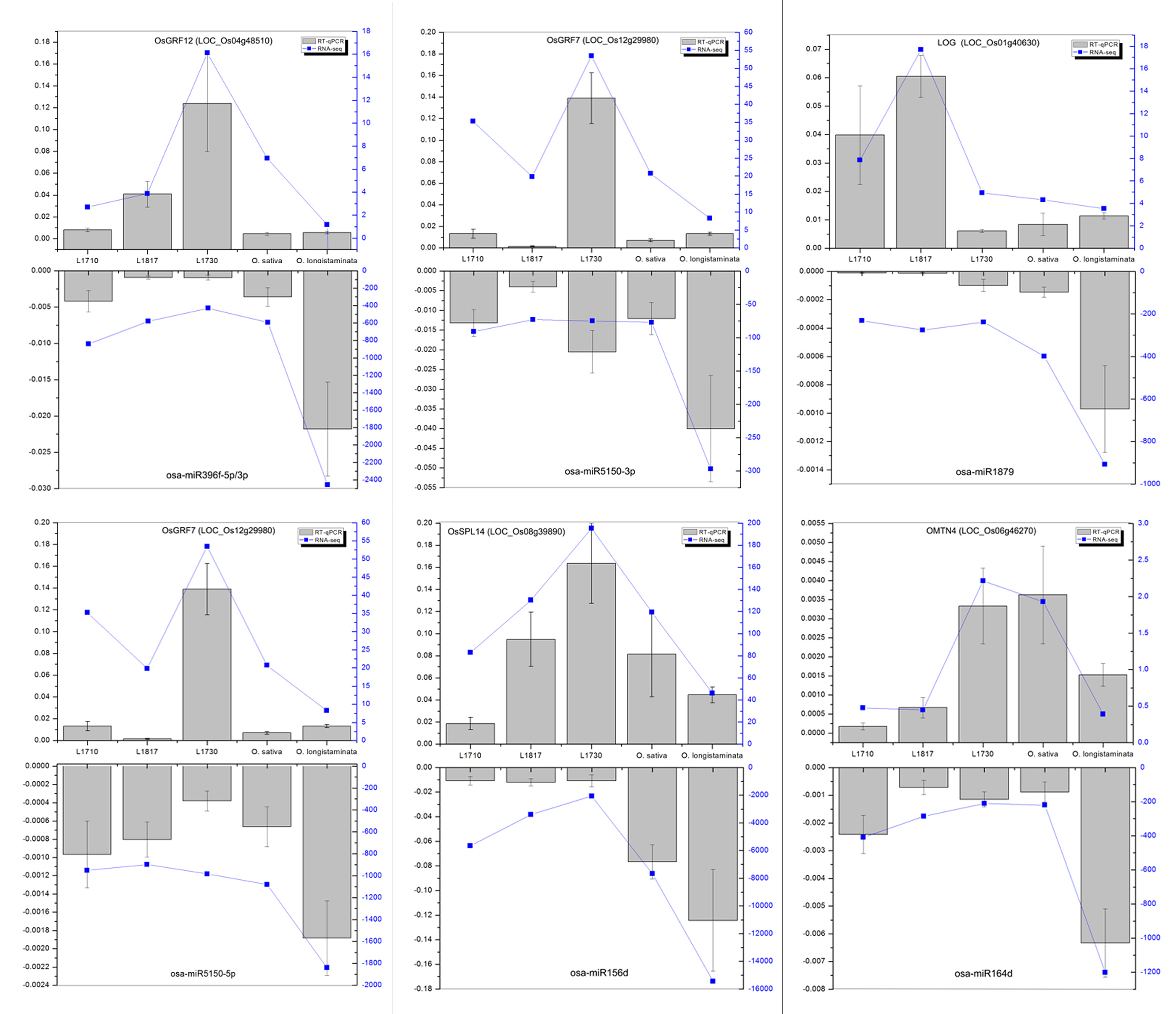

Supplement: S7 Fig — Actin1 and U6 snRNA are used as internal reference gene and small RNA, respectively. (TIF) [file pone.0184106.s007.tif]

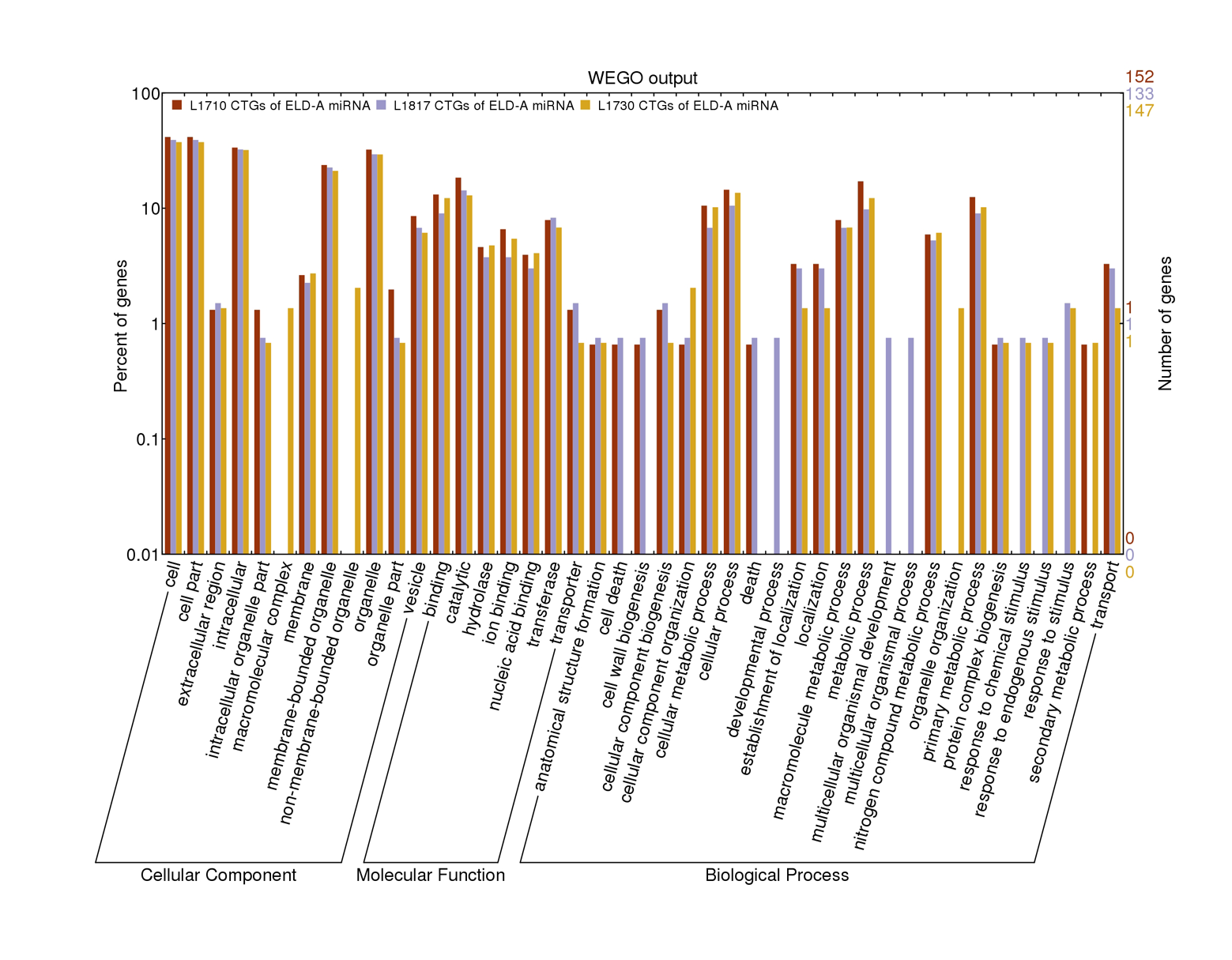

Supplement: S8 Fig — A stand for O. sativa. (TIF) [file pone.0184106.s008.tif]

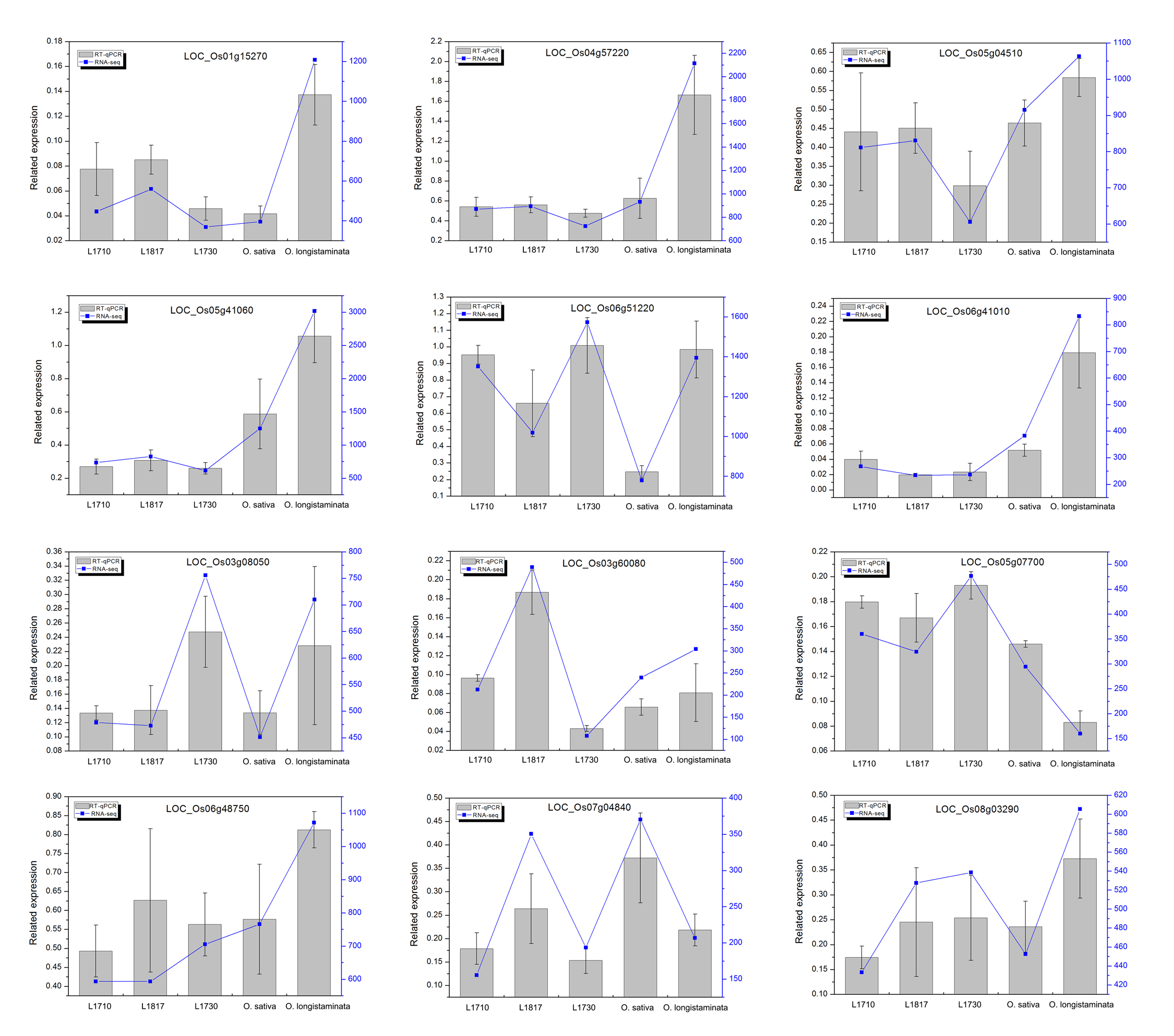

Supplement: S9 Fig — Actin1 is used as internal reference gene. The data of quantitative RT-PCR validation are expressed as the mean SD after normalization. Error bars indicate the standard deviation (±SD) of three replicates. Blue polylines are derived from RNA-Sqe data. (TIF) [file pone.0184106.s009.tif]

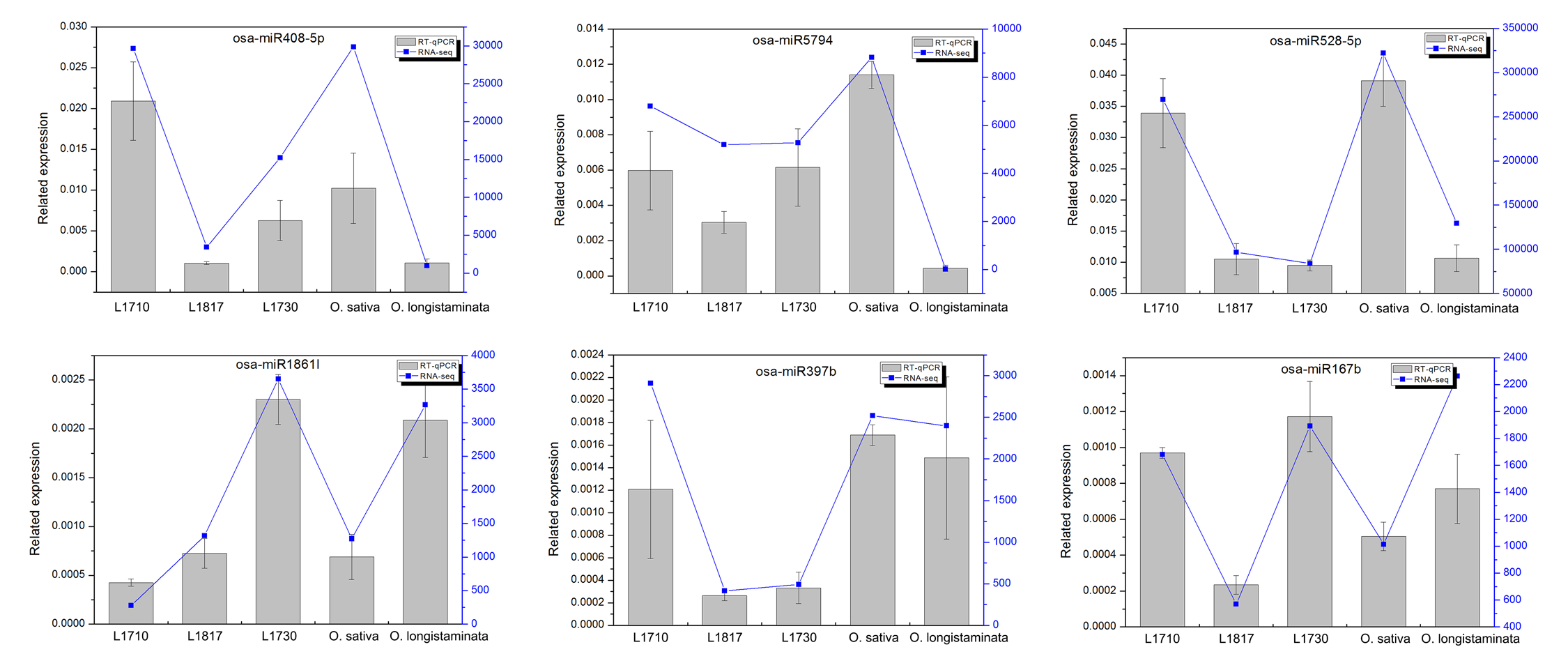

Supplement: S10 Fig — U6 snRNA is used as a reference small RNA. The data of real-time qPCR validation are expressed as the mean SD after normalization. Error bars indicate the standard deviation (±SD) of three replicates. Blue polylines are derived from RNA-Seq data. (TIF) [file pone.0184106.s010.tif]
