# Supplementary material for: Integrated analysis of mRNA and miRNA expression profiling in rice backcrossed progenies (BC2F12) with different plant height
Source: PLoS One. 2017 Aug 31;12(8):e0184106. doi: 10.1371/journal.pone.0184106 (PMC5578646; doi:10.1371/journal.pone.0184106)
Supplement: S3 Table — (DOCX) [file pone.0184106.s013.docx]

**S3 Table. Summary of mRNA sequencing read in five libraries.**

| Sample | Raw read | Clean read | Total mapped read | Gene expression |
| --- | --- | --- | --- | --- |
| L1710 | 12053564 | 11959710 (99.22%) | 10479672 (87.62%) | 26092 |
| L1817 | 12733597 | 12636297 (99.24%) | 11106158 (87.89%) | 26074 |
| L1730 | 11922151 | 11832864 (99.25%) | 10355542 (87.52%) | 26119 |
| *O. sativa* | 11877498 | 11792127 (99.28%) | 10345936 (87.74%) | 25363 |
| *O. longistaminata* | 11886247 | 11800115 (99.28%) | 9174319 (77.75%) | 25717 |
