# Supplementary material for: Integrated analysis of mRNA and miRNA expression profiling in rice backcrossed progenies (BC2F12) with different plant height
Source: PLoS One. 2017 Aug 31;12(8):e0184106. doi: 10.1371/journal.pone.0184106 (PMC5578646; doi:10.1371/journal.pone.0184106)
Supplement: S4 Table — (DOCX) [file pone.0184106.s014.docx]

**S4 Table. Distribution of mRNA sequence length in five libraries.**

| Abandance of gene length (bp) | Total number of genes  in five libaries | Percentage (%) |
| --- | --- | --- |
| 100-500 | 2323 | 7.24% |
| 500-1000 | 5615 | 17.50% |
| 1000-1500 | 6526 | 20.34% |
| 1500-2000 | 6262 | 19.52% |
| 2000-2500 | 4251 | 13.25% |
| ≥2500 | 7107 | 22.15% |
