# Supplementary material for: Integrated analysis of mRNA and miRNA expression profiling in rice backcrossed progenies (BC2F12) with different plant height
Source: PLoS One. 2017 Aug 31;12(8):e0184106. doi: 10.1371/journal.pone.0184106 (PMC5578646; doi:10.1371/journal.pone.0184106)
Supplement: S5 Table — (DOCX) [file pone.0184106.s015.docx]

**S5 Table. Number of considerably changed KEGG pathways in three progeny lines.**

| Comparison group | Number of all pathway | Number of up-regulated pathway | Number of down-regulated pathway | Number of significantly enriched pathway (P≤0.05) |
| --- | --- | --- | --- | --- |
| A-vs-L1710 | 88 | 77（65.3%） | 41（34.7%） | 16 (18.18%) |
| B-vs-L1710 | 117 | 70（59.3%） | 48（40.7%） | 25 (21.37%) |
| A-vs-L1817 | 89 | 67（57.3%） | 50（42.7%） | 13 (14.61%) |
| B-vs-L1817 | 117 | 59（50.4%） | 58（49.6%） | 28 (23.93%) |
| A-vs-L1730 | 94 | 52（44.8%） | 64（55.2%） | 18 (19.15%) |
| B-vs-L1730 | 114 | 39（33.6%） | 77（66.4%） | 36 (31.58%) |

A and B standing for *O. sativa* and *O. longistaminata*
