# Supplementary material for: Integrated analysis of mRNA and miRNA expression profiling in rice backcrossed progenies (BC2F12) with different plant height
Source: PLoS One. 2017 Aug 31;12(8):e0184106. doi: 10.1371/journal.pone.0184106 (PMC5578646; doi:10.1371/journal.pone.0184106)
Supplement: S6 Table — (DOCX) [file pone.0184106.s016.docx]

**S6 Table. The percentage of five categories in three progeny lines mRNA analysis.**

| Category | Additivity | ELD-A | ELD-B | Transgressive down-regulation | Transgressive up-regulation |  |
| --- | --- | --- | --- | --- | --- | --- |
| L1710 | 0.34% | 82.91% | 9.94% | 1.24% | 5.56% | |
| L1817 | 0.36% | 85.27% | 9.07% | 1.42% | 3.88% | |
| L1730 | 0.65% | 77.20% | 13.27% | 3.27% | 5.62% | |

A and B stand for *O. sativa* and *O. longistaminata*, respectively.
