# Supplementary material for: Integrated analysis of mRNA and miRNA expression profiling in rice backcrossed progenies (BC2F12) with different plant height
Source: PLoS One. 2017 Aug 31;12(8):e0184106. doi: 10.1371/journal.pone.0184106 (PMC5578646; doi:10.1371/journal.pone.0184106)
Supplement: S7 Table — (DOCX) [file pone.0184106.s017.docx]

**S7 Table. Distribution of small RNAs classes in five lines.**

| Small RNA class | L1710 | | L1817 | | L1730 | | *O. sativa* | | *O. longistaminata* | |
| --- | --- | --- | --- | --- | --- | --- | --- | --- | --- | --- |
|  | Unique sRNAs | Total sRNAs | Unique sRNAs | Total sRNAs | Unique sRNAs | Total sRNAs | Unique sRNAs | Total sRNAs | Unique sRNAs | Total sRNAs |
| total | 5578117 | 11451751 | 5152904 | 11379420 | 5769255 | 12165193 | 5245742 | 12022201 | 4472619 | 11228464 |
| miRNA | 4229 | 1285091 | 4203 | 762856 | 4540 | 757171 | 4277 | 1323683 | 3691 | 1274873 |
| rRNA | 37718 | 262394 | 46313 | 508589 | 32840 | 227188 | 32740 | 241367 | 45285 | 557021 |
| snRNA | 2502 | 7940 | 1705 | 4723 | 2036 | 6956 | 1871 | 5966 | 1648 | 4631 |
| snoRNA | 1295 | 1981 | 1208 | 1903 | 1101 | 1591 | 1008 | 1510 | 1199 | 1893 |
| repeat RNA | 1390791 | 3207132 | 1443335 | 3520057 | 1669959 | 4056382 | 1453937 | 3620994 | 797596 | 22253072 |
| tRNA | 17384 | 118171 | 17772 | 180058 | 18204 | 92711 | 17198 | 114581 | 12042 | 163428 |
| intron-sense | 87961 | 220669 | 95865 | 239846 | 104007 | 264280 | 96604 | 271113 | 52013 | 187467 |
| intron-antisense | 76300 | 142098 | 83629 | 164401 | 90385 | 177066 | 82030 | 170011 | 41673 | 94851 |
| exon-sense | 104742 | 179947 | 119506 | 192245 | 123895 | 207538 | 104642 | 187894 | 74613 | 152686 |
| exon-antisense | 64193 | 116469 | 67423 | 116162 | 78542 | 134387 | 65945 | 119959 | 40819 | 103717 |
| unannotated | 3791002 | 5909859 | 3271945 | 5688580 | 3643746 | 6239923 | 3385490 | 5965123 | 3402040 | 6434825 |
