# Supplementary material for: Integrated analysis of mRNA and miRNA expression profiling in rice backcrossed progenies (BC2F12) with different plant height
Source: PLoS One. 2017 Aug 31;12(8):e0184106. doi: 10.1371/journal.pone.0184106 (PMC5578646; doi:10.1371/journal.pone.0184106)
Supplement: S8 Table — (DOCX) [file pone.0184106.s018.docx]

**S8 Table. Distribution of abundance of miRNAs in three progeny lines and their parents.**

| Abundance of miRNAs | L1710 | | L1817 | | L1730 | | *O. sativa* | | *O. longistaminata* | |
| --- | --- | --- | --- | --- | --- | --- | --- | --- | --- | --- |
|  | Number of miRNA | Percentage（%） | Number of miRNA | Percentage（%） | Number of miRNA | Percentage（%） | Number of miRNA | Percentage（%） | Number of miRNA | Percentage（%） |
| 1-100 | 288 | 68.74% | 284 | 69.10% | 284 | 69.10% | 275 | 68.07% | 251 | 66.23% |
| 100-500 | 57 | 13.60% | 54 | 13.14% | 57 | 13.87% | 54 | 13.37% | 42 | 11.08% |
| 500-1000 | 15 | 3.58% | 14 | 3.41% | 15 | 3.65% | 13 | 3.22% | 16 | 4.22% |
| 1000-5000 | 30 | 7.16% | 40 | 9.73% | 34 | 8.27% | 32 | 7.92% | 43 | 11.35% |
| 5000-10000 | 13 | 3.10% | 7 | 1.70% | 8 | 1.95% | 17 | 4.21% | 6 | 1.58% |
| >10000 | 16 | 3.82% | 12 | 2.92% | 13 | 3.16% | 13 | 3.22% | 21 | 5.54% |
| Total | 419 | 100.00% | 411 | 100.00% | 411 | 100.00% | 404 | 100.00% | 379 | 100.00% |
