# Supplementary material for: Integrated analysis of mRNA and miRNA expression profiling in rice backcrossed progenies (BC2F12) with different plant height
Source: PLoS One. 2017 Aug 31;12(8):e0184106. doi: 10.1371/journal.pone.0184106 (PMC5578646; doi:10.1371/journal.pone.0184106)
Supplement: S9 Table — (DOCX) [file pone.0184106.s019.docx]

**S9 Table. The percentage of five categories in three progeny lines miRNA analysis.**

| Category | Additivity | ELD-A | ELD-B | Transgressive down-regulation | Transgressive up-regulation |  |
| --- | --- | --- | --- | --- | --- | --- |
| L1710 | 2.13% | 81.28% | 7.23% | 4.68% | 4.68% | |
| L1817 | 2.83% | 87.04% | 4.86% | 3.64% | 1.62% | |
| L1730 | 4.67% | 72.37% | 8.56% | 7.00% | 7.39% | |

A and B stand for *O. sativa* and *O. longistaminata*, respectively.
